# Survival differences between women and men in the non-reproductive cancers: Results from a matched analysis of the surveillance, epidemiology, and end-results program

**DOI:** 10.3389/fpubh.2022.1076682

**Published:** 2023-01-06

**Authors:** Felipe Andrés Cordero da Luz, Camila Piqui Nascimento, Eduarda da Costa Marinho, Pollyana Júnia Felicidade, Rafael Mathias Antonioli, Rogério Agenor de Araújo, Marcelo José Barbosa Silva

**Affiliations:** ^1^Center for Cancer Prevention and Research, Uberlandia Cancer Hospital, Umuarama, Uberlândia, Minas Gerais, Brazil; ^2^Laboratory of Tumor Biomarkers and Osteoimmunology, Department of Immunology, Institute of Biomedical Sciences, Federal University of Uberlandia, Umuarama, Uberlândia, Minas Gerais, Brazil; ^3^Medical Faculty, Federal University of Uberlandia, Umuarama, Uberlândia, Minas Gerais, Brazil

**Keywords:** solid cancer, sex, survival, cancer prevalence, non-reproductive cancer

## Abstract

**Background:**

Men with non-reproductive cancers have a discrepant outcome compared to women. However, they differ significantly in the incidence of cancer type and characteristics.

**Methods:**

Patients with single primary cancer who were 18 years or older and whose data were gathered and made accessible by the Surveillance, Epidemiology, and End Results (SEER) program were included in this retrospective analysis. Kaplan-Meier curves and Cox regression before and after propensity score matching were performed to analyze the risk survival by sex.

**Results:**

Among the 1,274,118 patients included [median (range) age, 65 year (18–85+) years; 688,481 (54.9%) male]. The median follow-up was 21 months (0–191). Substantial improvements in survival were observed for both sexes during the years of inclusion analyzed, with no difference between them, reaching a reduction of almost 17% of deaths in 2010, and of almost 28% in 2015, compared to 2004. The women had a median survival of 74 months and overall mortality of 48.7%. Males had a median survival of 30 months (29.67–30.33) with an overall mortality of 56.2%. The PSM showed a reduced difference (6 months shorter median survival and 2.3% more death in men), but no change in hazards was observed compared to the unmatched analysis [adjusted HR: 0.888 (0.864–0.912) vs. 0.876 (0.866–0.886) in unmatched].

**Conclusions:**

The discrepancy in survival between men and women is not explained only by the incidence of more aggressive and more advanced cancers in the former.

## 1. Introduction

Cancer is the leading cause of death among men and women, especially in developed countries (1). Cancer, like many diseases, can develop and progress in function of several factors (2). In this regard, gender is a considerable factor. Despite the increased incidence in women, men develop more cancers and have higher mortality than women (1, 3), in addition to the higher incidence of tumors, including non-reproductive tumors, of which men stand out (3). This difference in cancer risk between men and women may be attributable to a number of previously identified causes, like risky lifestyle—including greater alcohol consumption, smoking and delayed diagnosis -, impact of genetics, and sex hormones (2). All these factors affect men's metabolism, inflammation, and immunity, increasing their chance of getting and dying from cancer (4).

The recognition of this disparity has been more studied in the last decade after changes in the policies of preclinical and clinical research policies of the US National Institutes of Health (NIH) (4, 5). Studies have been developed using robust data sources to analyze sex differences in cancer risk and survival have been developed (6). In the United States, the Surveillance, Epidemiology, and End Results (SEER) Program provides an epidemiological database for researchers to analyze large cohorts of populations, which is a substantial resource to support new guidelines related to patient care, including cancer patients (7).

In view of the numerous scientific evidence, coming from large population bases, there is an increasing search to refine research, with appropriate design and analysis, in order to avoid failures that could result in statistically significant, but misleading, imprecise, or false conclusions (8).

In this sense, the present study aims to analyze the association of sex in the survival of the most prevalent solid cancers in both sexes after correcting the imbalance of potential confounding factors that can generate biases and/or spurious associations.

## 2. Material and methods

Retrospective observational study based on the Surveillance, Epidemiology and End Results (SEER) program [17 registries, Nov 2021 (2000–2019)] database, enrolling patients treated between 2004 and 2015. The database was analyzed with the ID 14659-Nov2021. Cancer-specific survival was measured as the main outcome.

### 2.1. Selection criteria

Patients 18 years of age or older diagnosed with a single primary cancer at the following sites were included: anus, anal canal and anorectal, appendix, rectosigmoid junction, rectum, sigmoid colon, descending colon, splenic flexure, hepatic flexure, transverse colon, ascending colon, cecum, esophagus, stomach, pancreas, kidney and renal pelvis, larynx, liver, lung, main bronchus, melanoma of the skin, brain, thyroid, trachea, and urinary bladder.

Patients were excluded according to the following criteria: no information on the reason for the absence of surgery; diagnosis only at autopsy or death certificate; follow-up time <1 day; stage 0 or occult cancer; lack of information on the exact site of cancer; and absence of sociodemographic information on race, median household income, type of housing region in the rural-urban continuum.

The rationale for the selection of patients for each analysis is depicted in Supplementary Figure 1. The syntax used in the SEERStat software for patient selection is found in the Supplementary material.

### 2.2. Classifications

The cancer staging systems used were the sixth and seventh editions of the AJCC for patients diagnosed between 2004–2009 and 2010–2015, respectively. The race identification used was restricted to Black, White and Other (American Indian/AK Native, Asian/Pacific Islander). The median household income adjusted for accumulated inflation for the year 2019 was used.

The staging, regardless of the edition of the AJCC system, has been reclassified as I, II, III, and IV. Unassigned or missing data regarding stage and histological grade were classified as NA (Not applied/not assigned); this category was not excluded from survival analyses or propensity score matching.

For survival, histologies (ICD-O-3) were grouped into an up to seven-category variable according to their relative frequency, in a decrease manner, for each site. An eighth variable was created grouping all remaining histologies. The codes of the first two categories are described in Supplementary Table 1.

For propensity score matching (PSM), histologies (ICD-O-3) were grouped into a three-category variable according to their relative frequency: most common, less common and rare. The codes of the first two categories are described in Supplementary Table 2, and the third category is composed of other histologies not described.

### 2.3. Statistical analysis

Statistical analyzes of normality (Kolmogorov-Smirnov), descriptive, crosstab (Pearson's χ^2^ and Cramer *V*-test), multinomial logistic regression, survival and PSM were performed on IBM SPSS v25.0. Kaplan-Meier plots and survival tables were performed on JAMOVI 2.2.5. The graphs representing the hazard ratios with a confidence interval of 95% were built in MS Excel. When appropriate, the significance level (α) was 0.05.

The Kaplan-Meier (KM) estimator was used to analyze the proportionality of risks based on curves and the Log-Rank test, as well as median survival, percentages, and censoring distributions. Only variables with proportionality of risks were included in Cox regression analysis. The proportionality of risks for continuous variables was evaluated by the correlation between the partial residues generated by the univariable Cox regression with the observation time of the analyzed outcome; the time dependence was assumed in the presence of correlation between these variables, visually analyzed by the scatter plot. In the case of the year variable, there was a correlation; its discrete nature impedes covariation by time (time-dependent Cox regression), and its categorized form was used instead of the quantitative form. Time dependence was also observed as a function of age, opting for a multicategorical variable instead of the continuous one.

Cox regression models were elaborated containing all the variables obtained (sex, year of diagnosis, age-14 categories -, race, median household income, rural-urban continuum, the reason for no cancer-direct surgery, histological grade, staging, cancer site, histological type-−8-category variable), except for the subgroup division variable, when applicable. Two-way and three-way interactions were included in the models, if the number of instances (number of patients) allowed for the complexity of the model (number of degrees of freedom). Two-way interactions between cancer site and histology and cancer site and age were entered into analyzes of the entire cohort and by sex, respectively. Three-way interactions between sex, age, and sites, and between sex, age, and histology were performed in full-cohort and site-wide analyses, respectively. In site and cancer analyses, it was possible to include the three-way interaction just before pairing. In analyses by year of diagnosis, it was possible to only the two-way interaction between site and histology; the other interactions were included only in the analysis of periods (2004–2009 and 2010–2015). In cases where it was not possible to include the three-way interaction, it was replaced by a respective two-way interaction that excludes age.

Significance level adjustment by the Bonferroni method was applied only to the variables of interest after the independence analysis in order interpret the prognostic value of these factors. Adjustment was performed by dividing the significance value by the number of possible pairs between variables included in each model. Unadjusted *p*-values were presented throughout the manuscript, except on express occasions.

For Cox regression analysis of sites with reduced sample number, it was used the bootstrapping technique with Bias Correction accelerated method to corroborate the findings; 1,000 resamples were performed. The interpretation of bootstrapping results was based on the analysis of confidence intervals, *p*-value, and bias for robust conclusions. Bootstrap *p*-values were reported in these cases only for multivariable models.

The directionality and location of the associations were evaluated by the analysis of standardized adjusted residuals. A Bonferroni correction was applied to adjust the level of significance to the amount of analysis by the interaction between rows and columns to obtain the significant associations. The interpretation of the effect size of the associations was adjusted by the degrees of freedom.

For logistic regression with the stage as a dependent, the parallel lines test was initially performed to assess the assumption of an ordinal regression. Due to the rejection of the null hypothesis of the parallel lines test (*p* < 0.05), a multinomial regression was performed. The proportionality/linearity with logits was analyzed by comparing the differences in the coefficients (βs) of the categories of the categorical variables and by the Box-Tidwell transformation for continuous variables. Continuous variables that break the assumption of significance in the insertion of the transformed variable [ln (variable)^*^variable] were categorized. Categories (levels) with overlapping confidence intervals or that break proportionality were collapsed with similar ones.

For PSM, the SPSS tool was used. Briefly, a 1:1 pairing without substitution was performed to match men and women (indicator group) according to the variables age, year of diagnosis (as categorical), race, median household income, rural-urban continuum classification, stage, histological type, cancer site, reason no cancer-directed surgery, and the AJCC edition used for staging. Additionally, the age variable of 14 categories and three-level histology variable were added to improve the matching. A tolerance of 0.000001 was established for matching based on the score generated by the matching logistic regression model. For Cox regression analysis after pairing, two models were developed: one covariate sex with the other variables, as abovementioned, and the other covariate sex with the score generated by the logistic regression of pairing.

## 3. Results

### 3.1. Patient characteristics

A total of 1,274,118 patients were selected, with a median age of 65 years (18–85+) and a median survival of 26 months (0–191). Of the entire cohort, 831,871 died, with 672,329 (80.8%) deaths attributed to cancer. Patients' characteristics are described separately by sexes in Table 1, which shows a considerable difference in the number of deaths and median survival between men and women.

**Table 1:** Socio-demographic and clinicopathological characteristics, by sex, of included patients diagnosed between 2004 and 2015 (*n* = 1,274,118).

**Variable**	**Female**	**Male**
*N*	585,637 (46.0%)	688,481 (54.0%)
Time of observation	35 months (range: 0–191)	21 months (range: 0–191)
**All-cause death**		
Alive	233,195 (39.8%)	209,052 (32.6%)
Dead	352,442 (60.2%)	479.429 (69.6%)
**Specific death**		
Alive/censored	300,496 (51.3%)	301,293 (43.8%)
Dead	285,141 (48.7%)	387,188 (56.2%)
Age	65 (range: 18–85+)	65 years (range: 18–85+)
**Age**		
≤ 25 years	8,498 (1.5%)	3,979 (0.6%)
26–30 years	10,052 (1.7%)	5,152 (0.7%)
31–35 years	14,263 (2.4%)	8,287 (1.2%)
36–40 years	18,908 (3.2%)	13,744 (2.0%)
41–45 years	26,671 (4.6%)	24,225 (3.5%)
46–50 years	39,331 (6.7%)	44,689 (6.5%)
51–55 years	52,272 (8.9%)	70,081 (10.3%)
56–60 years	59,453 (10.2%)	90,996 (13.2%)
61–65 years	65,269 (11.1%)	99,389 (14.4%)
66–70 years	66,311 (11.3%)	93,769 (13.6%)
71–75 years	65,187 (11.1%)	81,948 (11.9%)
76–80 years	61,999 (10.6%)	68,567 (10.0%)
81–84 years	43,273 (7.4%)	41,741 (6.1%)
≥85 years	54,150 (9.2%)	41,164 (6.0%)
**Race**		
White	448,876 (81.9%)	527,796 (82.3%)
Black	54,307 (9.9%)	62,483 (9.7%)
Other	44,960 (8.2%)	51,307 (8.0%)
**Household income**		
< $35K/year	10,168 (1.7%)	13,646 (2.0%)
≥$35 K/year and < $40 K/year	16,206 (2.8%)	21,645 (3.1%)
≥$40 K/year and < $45 K/year	26,998 (4.6%)	35,554 (5.2%)
≥$45 K/year and < $50 K/year	35,878 (6.1%)	45,307 (6.6%)
≥$50 K/year and < $55 K/year	50,532 (8.6%)	60,334 (8.8%)
≥$55 K/year and < $60 K/year	43,543 (7.4%)	52,728 (7.7%)
≥$60 K/year and < $65 K/year	98,052 (16.7%)	114,966 (16.7%)
≥$65 K/year and < $70 K/year	81,498 (13.9%)	93,668 (13.6%)
≥$70 K/year and < $75 K/year	50,373 (8.6%)	57,658 (8.4%)
≥$75 K/year	172,389 (29.4%)	192,975 (28.0%)
**Rural-urban continuum**		
Metropolitan (1 M+)	339,816 (58.0%)	388,250 (56.4%)
Metropolitan (250 K and < 1 M)	123,489 (21.1%)	143,979 (20.9%)
Metropolitan (< 250 K)	48,307 (8.2%)	58,764 (8.5%)
Non-metropolitan (adjacent)	42,982 (7.3%)	56,964 (8.3%)
Non-metropolitan (non-adjacent)	31,043 (5.3%)	40,524 (5.9%)
**Site**		
Anus, anal canal and anorectum	7,251 (1.2%)	4,294 (0.6%)
Appendix	3,372 (0.6%)	2,794 (0.4%)
Ascending colon	18,993 (3.2%)	15,479 (2.2%)
Brain	17,815 (3.0%)	23,372 (3.4%)
Cecum	23,233 (4.0%)	17,923 (2.6%)
Descending colon	5,105 (0.9%)	5,684 (0.8%)
Esophagus	6,204 (1.1%)	23,500 (3.4%)
Hepatic flexure	4,455 (0.8%)	4,210 (0.6%)
Kidney and renal pelvis	35,822 (6.1%)	57,602 (8.4%)
Larynx	3,739 (0.6%)	15,778 (2.3%)
Liver	13,194 (2.3%)	41,097 (6.0%)
Lung	163,006 (27.8%)	183,894 (26.7%)
Main bronchus	9,030 (1.5%)	10,785 (1.6%)
Melanoma of the skin	53,444 (9.1%)	64,156 (9.3%)
Pancreas	42,851 (7.3%)	43,336 (6.3%)
Rectosigmoid junction	9,462 (1.6%)	11,723 (1.7%)
Rectum	26,076 (4.5%)	35,047 (5.1%)
Sigmoid colon	24,684 (4.2%)	28,243 (4.1%)
Splenic flexure	2,775 (0.5%)	3,095 (0.4%)
Stomach	19,962 (3.4%)	30,478 (4.4%)
Thyroid	72,434 (12.4%)	20,902 (3.0%)
Trachea	109 (0.0%)	163 (0.0%)
Transverse colon	8,233 (1.4%)	7,611 (1.1%)
Urinary bladder	14,388 (2.5%)	37,315 (5.4%)
**Grade**		
Well differentiated (G1)	43,138 (7.4%)	39,644 (5.8%)
Moderately differentiated (G2)	132,463 (22.6%)	166,331 (24.2%)
Poorly differentiated (G3)	91,588 (15.6%)	131,112 (19.0%)
Undifferentiated/anaplastic (G4)	26,325 (4.5%)	42,040 (6.1%)
NA	292,123 (49.9%)	309,354 (44.9%)
**Stage**		
I	190,561 (32.5%)	185,688 (27.0%)
II	73,684 (12.6%)	91,335 (13.3%)
III	97,713 (16.7%)	123.085 (17.9%)
IV	159,558 (27.2%)	212,597 (30.9%)
NA	64,121 (10.9%)	75,776 (11.0%)
**Reason no cancer-directed surgery**		
Performed	339,079 (57.9%)	362,063 (52.6%)
Not recommended	213,598 (36.5%)	283,078 (41.1%)
Not recommended (contraindicated)	14,072 (2.4%)	19,229 (2.8%)
Recommended (refused)	7,763 (1.3%)	7,352 (1.1%)
Recommended (not performed for unknown reason)	8,139 (1.4%)	12,332 (1.8%)
Recommended (unknown if performed)	2,058 (0.4%)	2,980 (0.4%)
Not performed (died before recommendation)	928 (0.2%)	1,447 (0.2%)

NA, Not applied/not assigned.

### 3.2. Unbalanced data: Sex is a factor impacted by several confounders

Analyzes were performed to observe patterns and trends that may explain differences in survival between sexes, before performing survival analysis. Table 1 describes different frequencies of age groups, cancer sites, staging groups, and surgery decisions by sex. The significance of the observed difference was tested by contingency tables (Pearson's χ^2^ test) associated with the study of effect size (Cramer's *V*-test).

There was a significant association between the sex and age clusters (Pearson's χ^2^:23,147.50; Cramer's V_(1)_:0.135; *p* < 0.0005), cancer site (Pearson's χ^2^:76,809.54; Cramer's V_(1)_:0.246; *p* < 0.0005), and with the recommendation and performance of surgery according to sex (Pearson's χ^2^:4,149.46; Cramer's V_(1)_:0.057; *p* < 0.0005). There were also significant associations between sex and histology per site, and a positive association of both sexes with some histology per cancer site. Moreover, associations between age clusters and cancer sites were observed per sex. The Supplementary results describes the details and association of age cluster and sex by cancer site.

Regarding stage, although the female sex was positively associated with stage I cancer (std. res.: 68.7) and the male sex mainly with stage IV cancer (std. res.: 45.0), this association was weak (Pearson's χ^2^:5,128.74, *p* < 0.0005) with a negligible effect size (Cramer's V_(1)_:0.063, *p* < 0.0005).

However, other associations are not directly perceived with sex, which could be potential confounding effects. While no expressive numeric differences were observed between males (Pearson's χ^2^:403,888.37; Cramer's V_(4)_:0.383; *p* < 0.0005) and females (Pearson's χ^2^:385,634.62; Cramer's V_(4)_:0.406; *p* < 0.0005), there was a very strong association between site and stage (Pearson's χ^2^:788,214.79, *p* < 0.0005) with a large effect size (Cramer's V_(4)_:0.393, *p* < 0.0005) (Supplementary Table 3), which prevailed even when excluding brain cancer in the analysis (Pearson's χ^2^:439,064.97; Cramer's V_(3)_:0.298; *p* < 0.0005).

### 3.3. Trends over time according to sex

**Figure 1:**
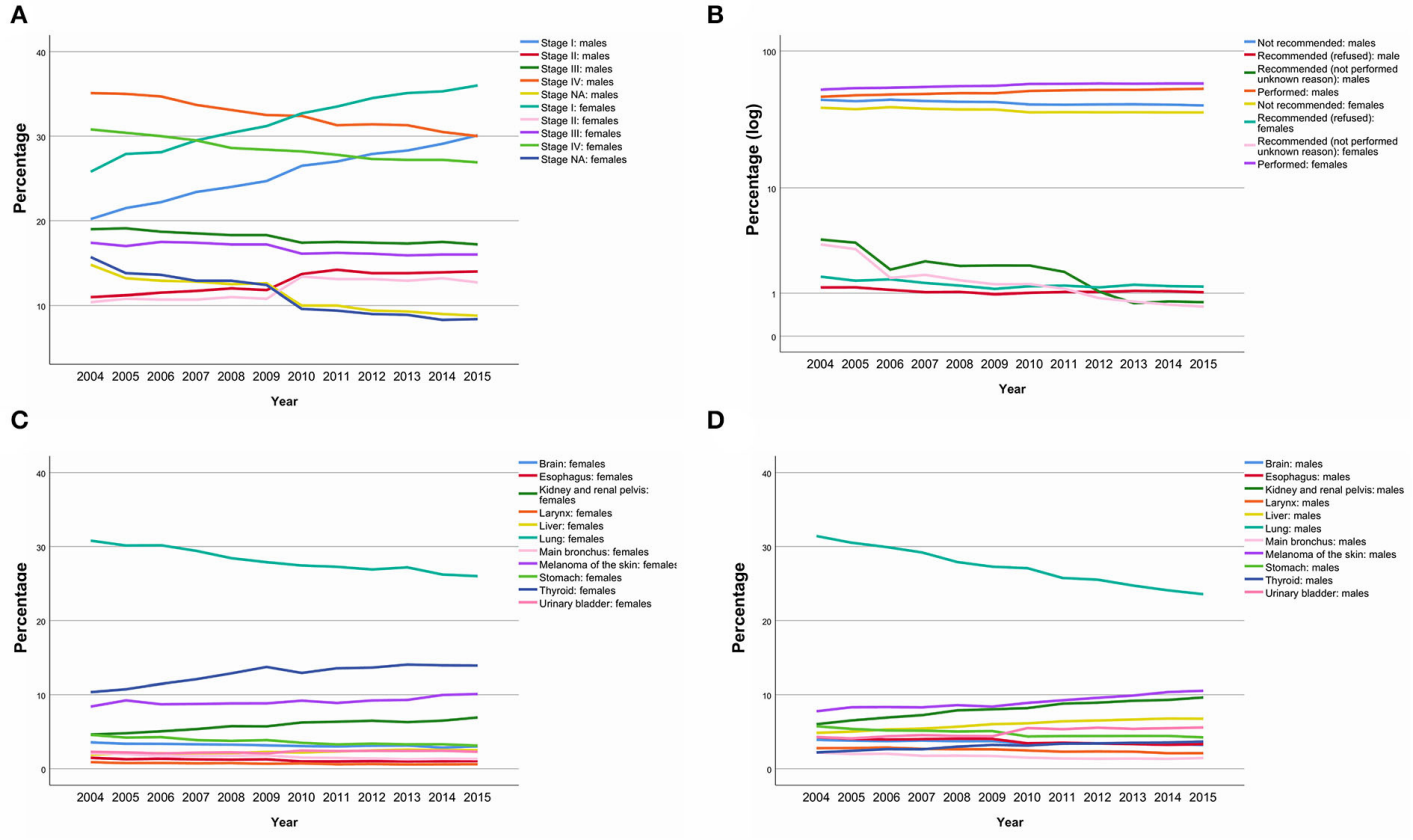
Stage, surgery performance and cancer site prevalence trends over time by sex. Stage at diagnosis **(A)**. Main surgery performances changes **(B)**. Main cancer site changes in females **(C)**. Main cancer site changes in males **(D)**.

Despite the differences described above, it is possible to observe a consistent increase in cancer diagnosis in earlier stages (Figure 1A). Additionally, an increase in the performance of surgeries, with a reduction of no recommendations, refusals, and lack of surgery performance, was observed over time (Figure 1B). There is also a reduction in the prevalence of respiratory, brain, thyroid, and stomach cancers, with a compensatory increase in melanomas of the skin, liver, and urinary system (Figures 1C, D). There is no apparent difference in trends by sex.

### 3.4. Regression models: Sex as an independent factor of poor prognostic variables

Multinomial logistic regression models were used to confirm the impact of sex and year on staging and surgery performance.

Using the “surgery performed” classification as a reference and adjusting for age, year of diagnosis, race, household income, rural-urban continuum, site of cancer, stage, and histological grade, men are more likely to die before surgery recommendation [OR: 1.342 (1.232–1.462), *p* < 0.0005], being contraindicated for surgery [OR: 1.188 (1.159–1.218), *p* < 0.0005], and being recommended but not performing surgery [OR: 1.291 (1.252–1.332), *p* < 0.0005], but are less likely to refuse surgery [OR: 0.949 (0.917–0.982), *p* = 0.003], compared to females.

For analysis of stage predictive classification, only patients with stage were included (*n* = 1,134,221). Using the “stage I” classification as a reference and adjusting for age, year of diagnosis, race, household income, rural-urban continuum, cancer site, and histological grade, men are more likely to be classified as “stage II” [OR: 1.173 (1.158–1.189), *p* < 0.0005], III [OR: 1.226 (1.211–1.240), *p* < 0.0005], and IV [OR: 1.283 (1.269–1.298), *p* < 0.0005], compared to females.

### 3.5. The effect of sex on survival: Balancing the factors reduces the gap

The association of cancer site with stage, in addition to the association of sex with age and cancer site, the excess of stage IV in men and stages trends over time can generate highly biased results in survival analyses. For computational limitations, the PSM was performed separately by tumor sites and then merged after matching. Due to the small number of patients with tracheal cancer, there was no matching. Cancer-specific survival was used instead of overall survival to suppress the longer life expectancy bias of women compared to men (9).

After matching, there was no further association between sex and site (Pearson's χ^2^: 0; *p* = 1.000; Cramer's V_(1)_: 0.000), but other associations, although negligible, still exist (Supplementary results). Importantly, the difference in survival by site of the cancer remained practically the same after pairing as before pairing (Supplementary Table 4 and Supplementary Figure 2).

**Figure 2:**
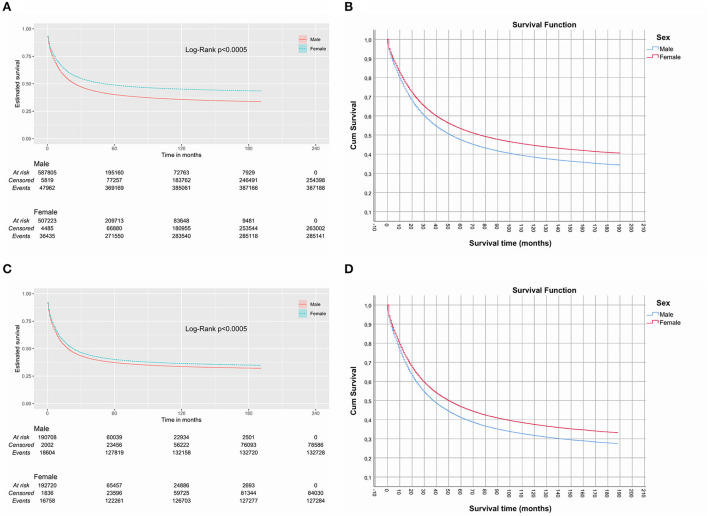
Kaplan-Meier and adjusted plots of cancer specific survival. Before **(A, B)** and after **(C, D)** matching.

By the Kaplan-Meier and Log-Rank comparison (χ^2^: 8,335.54; *p* < 0.0005) it was possible to observe significantly higher survival of female patients in the entire cohort (Figure 2A) and in almost all cancer sites (Supplementary Table 5). Female patients had a median survival of 74 months, compared to 30 months (29.67–30.33) of the male counterpart, which had an excess of 7.5% of deaths in relation to women (female: 48.7%; male: 56.2%). This difference remained after adjustment by other variables in multivariable Cox regression [adjusted HR: 0.876 (0.866–0.886), *p* < 0.0005] (Figure 2B). The 1-, 3-, 5-year, and longer survival tables, with the number at risk, are described in Supplementary Table 6.

After matching by propensity score (*n* = 441,916), this difference in median survival of 44 months was reduced to only 6 months [female: 29 months (28.38–29.62); male: 23 months (22.60–3.40)], although this difference was still significant (Log-Rank χ^2^: 431.13; *p* < 0.0005) (Figure 2C). The excess death in men compared to women dropped to 2.3% (female: 56.1%; male: 58.4%). Again, this difference remained after adjustment by other variables in multivariable Cox regression [adjusted HR: 0.888 (0.864–0.912), *p* < 0.0005] (Figure 2C). The 1-, 3-, 5-year and longer survival tables, with the number at risk, are described in Supplementary Table 7.

**Figure 3:**
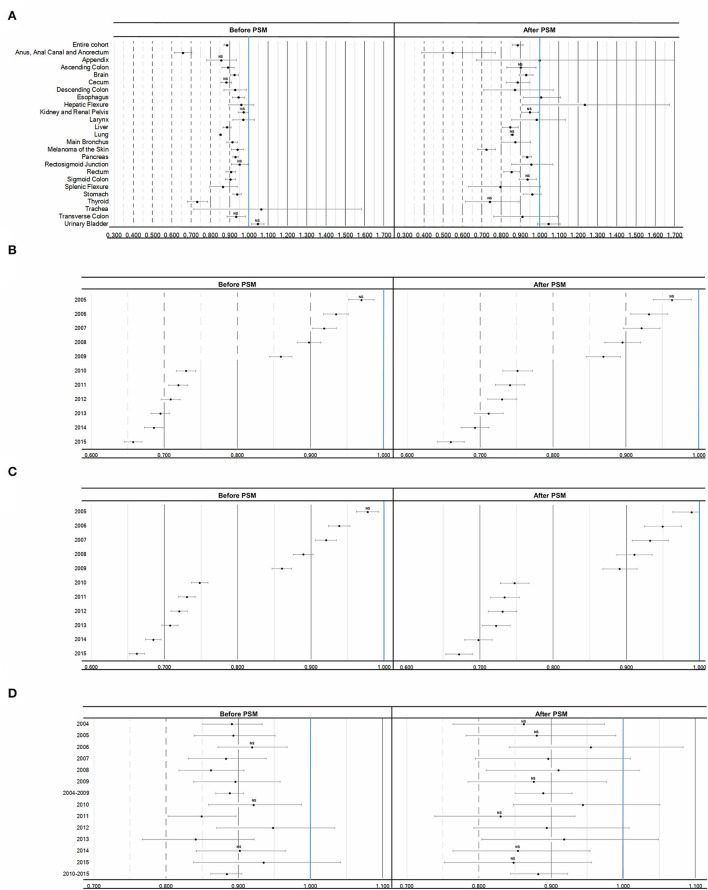
Adjusted Hazard Ratios (95% CI) of the effect of sex on cancer specific survival. **(A)** Female association referenced to male before and after matching in the entire cohort and per cancer site. **(B)** Cancer specific survival by year referenced to 2004 in female patients before and after matching. **(C)** Cancer specific survival by year referenced to 2004 in male patients before and after matching. **(D)** Female association referenced to male before and after matching by year or period. Legend: NS, not statistically significant after Bonferroni adjustment of *p*-value.

The site was analyzed separately because the association of sex with survival may vary depending on the type of cancer. The effect of sex in multivariable analyzes before and after matching lost its significance in the *p*-value adjustment for some situations (Figure 3A; Supplementary Tables 8, 9).

A consistent increase in cancer-specific survival was observed over time for both sexes (Figures 3B, C; Supplementary Tables 10, 11), with no difference in the trend by sex. Despite the absence of a difference in the association between sex and survival in some years, in general, a positive association between women and survival was observed, including analyzes by periods (2004–2009 and 2010–2015) (Figure 3D; Supplementary Tables 12, 13), which could imply some bias due to the change in the staging system. KM and adjusted Cox regression curves (Supplementary Figures 3, 4), as well as 1-, 3-, 5-year and longer survival tables, with number at risk, are described in Supplementary Tables 14, 15.

## 4. Discussion

The present study showed the difference in non-reproductive cancer-specific survival (CSS) that exists between men and women using data made available by the SEER Program, spanning the recent decade of 2004–2015. As expected, women have a lower risk of dying than men (3, 6, 10–14).

We confine ourselves to including the most frequent non-sexual solid cancers by systems, excluding sarcomas, and excluding some with potential bias, with head and neck cancer being a particular case. Patients whose etiology resides in HPV infection have a somewhat better prognosis for some sites compared to those HPV-negative (15–18), mainly in women (18), with the prevalence of oral HPV infection being higher in men than in women (19). Additionally, this factor became available in SEER only from 2010 (7). Thus, unlike these studies, we did not include head and neck cancers.

It was observed an increase of early diagnosis (1, 3) and a decrease in mortality over the years (1, 14). Additionally, it was possible to observe a reduction in the prevalence of thyroid and lung cancers (1), along with a higher prevalence of non-reproductive cancers in men compared to women (4).

This study showed the high heterogeneity between them, which potentially acts as a confounder and even collider with the effect of biological sex on the outcome of many solid tumors. Although there were not such strong associations between sex and other variables, indirect associations given the particularity of the most frequent cancers in each sex, have very different results. For example, women were positively associated with thyroid cancer, which is much more common in them and has a better prognosis (1, 12, 20, 21), while men were positively associated with cancers of the esophagus, liver, stomach and urinary bladder, which have a poor prognosis (1, 6, 11–13). Thus, most of the discrepancy observed in survival by sex is due to characteristics of incidence and stage at diagnosis, and men are affected by more aggressive types of cancer and are diagnosed later.

In this study, it was observed that men and women differ in age at diagnosis, observing some bimodal associations depending on the cancer, that is, for the same sex there is an association with extremes of age (early and later). More importantly, a positive association was observed between female sex and earlier age in cancers that are screenable, such as thyroid and colorectal, as well as others, mainly melanoma of the skin. In this regard, a potential undesirable effect is the lead time bias, which occurs primarily in screenable cancers, resulting in a prolongation of survival due to overdiagnosis of cancers with a very good prognosis (22, 23). Although there are sophisticated statistical methods to correct this problem (22, 23), match resulted in a vast reduction of this disparity, but without completely eliminating it.

Such data imbalances can impact the analyses, even when adjusting for other variables. In the presence of excess confounders, which can also lead to a collider effect, the effect of the variable of interest is suppressed, which is very common in large database-based observational studies (24). Additionally, it is very common to find spurious associations and correlations in massive database analysis simply because they have a high number of instances (25). In this sense, this study presents something new in relation to the others. In addition to focusing exclusively on non-reproductive cancers and using a clinical practice staging system, this series of SEER-based analyzes were performed by adjusting for several confounders after matching them, which may subvert the association of sex with survival.

Although the average risk (Hazard Ratio - HR) remained the same after match, the effect of these imbalances was clear in reducing the median survival, with a wide narrowing in the difference between men and women after matching (from 74 to 6 months). Additionally, HRs and their confidence intervals underwent substantial changes, especially in cancer site analyses. For example, in prematching analyzes, it was possible to observe a lack of significance with wide confidence intervals in the association of sex with survival at several tumor sites but turned significant after adjusting for other factors. In addition, there was a loss of significance of this association for some sites after matching.

Due to all the above-mentioned issues, the results after pairing are more reliable, a fact that is one of the main distinguishing features of the other studies, thus demonstrating a better outcome for women than men, but, in relation to the sites, there is no difference in colon cancer (except ascending and sigmoid), esophagus, larynx, stomach, and urinary bladder, different from that observed by others (3, 11, 12). Additionally, there was a loss of significance after correction of the *p*-value, such as for some colon sites and thyroid, despite not being a common practice in survival analysis. This could represent both an advance in cancer management and a correction of confounders' and colliders' effect-laden data.

However, disregarding the *p*-value correction, the discrepancy in survival by sex for some sites is notable, being very large in cases such as the anus, anal canal and anorectal, melanoma, and thyroid. Even observing that the male sex proved to be an independent factor of association with later stages, not performing surgical intervention or dying before surgery, which is in line with a riskier lifestyle and a lower demand for medical care by men (2, 4, 26), it does not seem plausible to attribute such a discrepancy to these factors alone.

In fact, women perform much better in cancer progression. Going from the molecular basis, where there are even sexual genetic patterns for some cancers (27), through biochemical, hormonal, immunological, anatomical, behavioral, exposition to risk factors, and even response to therapies, women show an advantage over men in general (26, 28–32). Particularly speaking of the higher incidence of esophageal, stomach and liver cancers in men, although the main causes are current or greater consumption of alcohol and cigarettes (33–39), as well as a rate of infection by hepatitis B and C viruses (34, 40), some important biological factors must be considered. For example, among current smokers, the risk of developing stomach cancer is higher in men (33), and men infected with the Hepatitis B virus are more likely to die from liver cancer than women infected with the same virus (34).

The question regarding therapy has an important sex-specific survival effect as the dosage is not usually adjusted proportionally to the lean mass of the male body (26). Furthermore, women respond better to lipophilic drugs, while men respond better to hydrophilic drugs (26). On the other hand, the development and incorporation of new therapies into treatment regimens, such as immunotherapies (41–43) and small molecule drugs (44–46) for melanoma and lung cancer, has been shown to be a paradigm shift in cancer survival. However, this is a limitation of studies such as the present one (3, 11, 12, 14), since there was no access to adherence and the type of therapy to which patients were submitted.

Added to this limitation, this study has other limitations. For example, specific survival was used instead of overall survival to reduce the impact of the difference in life expectancy between men and women that would reproduce this outcome (9). On the other hand, specific survival tends to be underestimated in relation to relative survival (47), but the difficulty in obtaining relative survivals for certain subgroups (47). In addition, there are difficulties in the implementation of statistical methods to correct such baseline survivals in Cox regression models (48), make its implementation unfeasible.

Also, due to the complexity of the models, it was not possible to implement the time-dependent Cox regression in certain analyses, such as the entire cohorts. Despite this, simpler models showed similar results for the purpose of the study (not shown).

Furthermore, due to the information available in the SEER Program, the inclusion of only patients with identification of urban contingency led to the exclusion of all those diagnosed in the states of Alaska and Hawaii, leading to an underrepresentation of certain characteristics of the population.

Similar population studies considering types of systemic treatments, comorbidities and other risk factors, such as the amount of alcohol and tobacco used, are needed to analyze whether the difference in survival between men and women narrows further or widens.

In summary, although it is not possible to determine the causes of gender disparities in cancer survival, the findings seek to encourage further studies that reduce the impact of the male on cancer prognosis.

## 5. Conclusions

Men have a higher prevalence of more aggressive non-reproductive cancers and more advanced stages, negatively impacting survival. However, in general, women with the same characteristics have longer survival.

## Data availability statement

The original contributions presented in the study are included in the article/Supplementary material, further inquiries can be directed to the corresponding author.

## Ethics statement

Ethical review and approval was not required for the study on human participants in accordance with the local legislation and institutional requirements. Written informed consent for participation was not required for this study in accordance with the national legislation and the institutional requirements.

## Author contributions

FL contributed to the conception and design of the study. FL, RAA, and MS contributed to data collection and quality control. All authors drafted the paper and interpreted the results. All authors contributed to data interpretation and rewriting the paper, reviewed and approved the final version, and had full access to all the data and were responsible for the decision to submit the manuscript.
