# Supplementary material for: Survival differences between women and men in the non-reproductive cancers: Results from a matched analysis of the surveillance, epidemiology, and end-results program
Source: Front Public Health. 2023 Jan 6;10:1076682. doi: 10.3389/fpubh.2022.1076682 (PMC9853080; doi:10.3389/fpubh.2022.1076682)
Supplement: Supplementary file 1 [file Data_Sheet_1.docx]

Supplementary Material

# Supplementary Data

## Supplementary Methods

*Selection criteria*

The syntax used in the SEERStat software is as follows:

{Race and Age (case data only).Age recode with single ages and 85+} != '00 years','01 years','02 years','03 years','04 years','05 years','06 years','07 years','08 years','09 years','10 years','11 years','12 years','13 years','14 years','15 years','16 years','17 years'

AND {Site and Morphology.Behavior code ICD-O-3} = 'Malignant'

AND {Multiple Primary Fields.Sequence number} = 'One primary only'

AND {Race, Sex, Year Dx.Year of diagnosis} = '2004','2005','2006','2007','2008','2009', '2010', '2011', '2012', '2013', '2014', '2015'

AND {Site and Morphology.Site recode ICD-O-3/WHO 2008} = ' Esophagus',' Stomach',' Cecum',' Appendix',' Ascending Colon',' Hepatic Flexure',' Transverse Colon',' Splenic Flexure',' Descending Colon',' Sigmoid Colon' ,' Rectosigmoid Junction',' Rectum',' Anus, Anal Canal and Anorectum',' Liver',' Pancreas',' Larynx',' Lung and Bronchus',' Trachea, Mediastinum and Other Respiratory Organs',' Melanoma of the Skin',' Urinary Bladder',' Kidney and Renal Pelvis',' Brain',' Thyroid'

AND {Site and Morphology.Site recode ICD-O-3/WHO 2008} != ' Large Intestine, NOS'

AND {Site and Morphology.Primary Site - labeled} != 'C38.1-Anterior mediastinum','C38.2-Posterior mediastinum','C38.3-Mediastinum, NOS','C38.8-Overlapping lesion of heart, mediastinum and pleura','C39.0-Upper respiratory tract, NOS','C39.8-Overlapping lesion of respiratory system','C39.9-Ill-defined sites within respiratory system'

AND {Therapy.Reason no cancer-directed surgery} != 'Unknown; death certificate; or autopsy only (2003+)'

AND {Cause of Death (COD) and Follow-up.SEER cause-specific death classification} != 'Dead (missing/unknown COD)','N/A not seq 0-59'

AND {Cause of Death (COD) and Follow-up.SEER other cause of death classification} != 'N/A not seq 0-59'

AND {Cause of Death (COD) and Follow-up.Survival months flag} = 'Complete dates are available and there are more than 0 days of survival'

AND {Race, Sex, Year Dx.Race recode (White, Black, Other)} = ' White',' Black',' Other (American Indian/AK Native, Asian/Pacific Islander)'

AND {County attributes - Time Dependent.Median household income inflation adj to 2019} != 'Unknown/missing/no match/Not 1990-2018'

AND {County attributes - Time Dependent.Rural-Urban Continuum Code} != 'Unknown/missing/no match (Alaska or Hawaii - Entire State)','Unknown/missing/no match/Not 1990-2018'

Stage 0 and occult disease were manually excluded according to the current edition of the AJCC staging manual for the year of diagnosis.

## Supplementary Results

*Unbalanced data: sex is a factor impacted by several confounders*

While a positive association was observed between male sex and age ranges 51-75 years, a positive association was observed between female sex and age ranges ≤40 years (std. res. – std. res.: 98.2), 41-45 years (std. res.: 29.7), 76-80 years (std. res.: 11.6) and >80 years (std. res.: 74.1).

There was a significant association between sex and cancer site. Male sex was positively associated with cancer in the brain (std. res.: 11.2), esophagus (std. res.: 87.8), kidney and renal pelvis (std. res.: 48.6), larynx (std. res.: 75.7), liver (std. res.: 103.5), rectum (std. res.: 16.8), stomach (std. res.: 29.4) and urinary bladder (std. res.: 84.5).

On the other hand, female sex was positively associated with cancer in the anus, anal canal and anorectum (std. res.: 36.5), appendix (std. res.: 13.8), ascending colon (std. res.: 34.5), cecum (std. res.: 43.4), lung (std. res.: 14.2), pancreas (std. res.: 22.9), thyroid (std. res.: 201.5) and transverse colon (std. res.: 15.2).

There was a notable association of female sex with surgeries performed in females (std. res.: 60.1).

There was also significant associations between sex and histology per sites, with emphasis on associations in histologies of cancer in the anus, anal and anorectal canal (Pearson’s χ2: 408.03; Cramer’s V_(1)_: 0.188; p<0.0005), appendix (Pearson’s χ2: 126.07; Cramer’s V_(1)_: 0.143; p<0.0005), esophagus (Pearson’s χ2: 1,752.08; Cramer’s V_(1)_: 0.243; p<0.0005), kidney and renal pelvis (Pearson’s χ2: 1,049.58; Cramer’s V_(1)_: 0.106; p<0.0005), liver (Pearson’s χ2: 1,295.06; Cramer’s V_(1)_: 0.154; p<0.0005), lung (Pearson’s χ2: 5,846.60; Cramer’s V_(1)_: 0.130; p<0.0005), main bronchus (Pearson’s χ2: 652.98; Cramer’s V_(1)_: 0.182; p<0.0005), rectum (Pearson’s χ2: 908.86; Cramer’s V_(1)_: 0.122; p<0.0005), stomach (Pearson’s χ2: 1,699.85; Cramer’s V(1): 0.184; p<0.0005), thyroid (Pearson’s χ2: 940.87; Cramer’s V_(1)_: 0.100; p<0.0005), and urinary bladder (Pearson’s χ2: 1,157.73; Cramer’s V_(1)_: 0.150; p<0.0005). It was observed.

It was observed a positive association of female sex and the ICD-O-3 histology 8083/3 (std. res.: 9.2) of the anus, anal and anorectal canal; 8000/3 (std. res.: 8.6) of the brain; 8000/3 (std. res.: 5.3), 8041/3 (std. res.: 5.2), 8070/3 (std. res.: 34.8), 8071/3 (std. res.: 9.1), 8720/3 (std. res.: 5.1) of the esophagus; 8120/3 (std. res.: 10.5) of the kidney and renal pelvis; 8200/3 (std. res.: 5.0) of the larynx; 8010/3 (std. res.: 10.9), 8010/3 (std. res.: 6.0), 8140/3 (std. res.: 15.2), 8160/3 (std. res.: 21.4), 8174/3 (std. res.: 5.4), 8240/3 (std. res.: 6.1), 8246/3 (std. res.: 7.4), 8890/3 (std. res.: 5.6), 9133/3 (std. res.: 5.4) of the liver; 8000/3 (std. res.: 11.1), 8041/3 (std. res.: 9.6), 8240/3 (std. res.: 29.3), 8250/3 (std. res.: 21.6), 8253/3 (std. res.: 5.4), 8255/3 (std. res.: 13.0), 8260/3 (std. res.: 5.3), 8480/3 (std. res.: 5.3), 8550/3 (std. res.: 13.3) of the lung; 8041/3 (std. res.: 12.7), 8140/3 (std. res.: 5.5), 8240/3 (std. res.: 4.9) of the main bronchus; 8743/3 (std. res.: 17.8), 8744/3 (std. res.: 6.1) of melanoma; 8000/3 (std. res.: 12.5), 8010/3 (std. res.: 8.6), 8470/3 (std. res.: 6.3) of pancreas; 8070/3 (std. res.: 17.6), 8072/3 (std. res.: 6.3), 8083/3 (std. res.: 6.7), 8240/3 (std. res.: 14.2) of the rectum; 8000/3 (std. res.: 5.7), 8010/3 (std. res.: 6.4) of the sigmoid colon; 8000/3 (std. res.: 6.1), 8142/3 (std. res.: 5.0), 8145/3 (std. res.: 5.2), 8240/3 (std. res.: 22.5), 8490/3 (std. res.: 18.4), 8936/3 (std. res.: 13.9) of the stomach; 8340/3 (std. res.: 11.9), 8341/3 (std. res.: 7.8) of the thyroid; 8010/3 (std. res.: 6.7), 8070/3 (std. res.: 20.5), 8071/3 (std. res.: 11.0), 8120/3 (std. res.: 6.5), 8140/3 (std. res.: 6.8), 8310/3 (std. res.: 5.5) of the urinary bladder.

For the other hand, it was observed a positive association of male sex and the ICD-O-3 histology 8051/3 (std. res.: 8.0), 8480/3 (std. res.: 6.1) the anus, anal and anorectal canal; 8140/3 (std. res.: 33.2), 8490/3 (std. res.: 6.3) of the esophagus; 8260/3 (std. res.: 24.3), 8318/3 (std. res.: 4.9) of kidney and renal pelvis; 8170/3 (std. res.: 31.7) of the liver; 8012/3 (std. res.: 7.8), 8046/3 (std. res.: 15.2), 8070/3 (std. res.: 54.7), 8071/3 (std. res.: 15.9), 8072/3 (std. res.: 5.5) of the lung; 8070/3 (std. res.: 20.1) of the main bronchus; 8721/3 (std. res.: 11.2), 8742/3 (std. res.: 19.2) of melanoma; 8140/3 (std. res.: 10.1), 8550/3 (std. res.: 6.5) of the pancreas; 8140/3 (std. res.: 16.7) of the rectum; 8140/3 (std. res.: 30.2) of the stomach; 8010/3 (std. res.: 5.3), 8021/3 (std. res.: 10.7), 8290/3 (std. res.: 6.4), 8330/3 (std. res.: 8.4), 8347/3 (std. res.: 5.5), 8510/3 (std. res.: 15.5) of the thyroid; 8130/3 (std. res.: 20.8) of the urinary bladder.

The association of earlier age and sex was specific to cancer sites. There was a positive association between earlier ages and male sex for cancers of the anus, anal and anorectum (≤55 years), ascending colon (≤70 years), brain (≤65 years), cecum (≤70 years), esophagus (≤70 years), hepatic flexure (≤65 years), pancreas (≤70 years), and transverse colon (≤70 years). On the other hand, there was a positive association between earlier ages and the female sex in relation to cancers of the descending colon (≤40 years), kidney and renal pelvis (≤35 years), larynx (≤55 years), liver (≤40 years), lung (≤50 years), melanoma of the skin (≤50 years), rectosigmoid junction (≤40 years), rectum (≤40 years), sigmoid colon (≤45 years), stomach (≤45 years), and thyroid (≤45 years).

The association of medium age and sex was also specific to cancer sites, and only for male sex. There was a positive association between medium ages and male sex for cancers of the descending colon (>55 and ≤70 years), kidney and renal pelvis (>40 and ≤65 years), larynx (>55 and ≤70 years), liver (>45 and ≤65 years), lung (>51 and ≤70 years), main bronchus (>55 and ≤70 years), rectosigmoid junction (>50 and ≤70 years), rectum (>45 and ≤70 years), sigmoid colon (>55 and ≤75 years), splenic flexure (>55 and ≤70 years), stomach (>45 and ≤75 years), and urinary bladder (>50 and ≤75 years).

The association of latter age and sex was also specific to cancer sites. There was a positive association between latter ages and male sex for cancers of the melanoma of the skin (>50 years) and thyroid (>50 years). On the other hand, there was a positive association between latter ages and the female sex in relation to cancers of the anus, anal and anorectum (>60 years), ascending colon (>75 years), brain (>70 years), cecum (>75 years), descending colon (>80 years), esophagus (>70 years), hepatic flexure (>75 years), kidney and renal pelvis (>70 years), liver (>65 years), lung (>75 years), main bronchus (>75 years), pancreas (>75 years), rectosigmoid junction (>75 years), rectum (>75 years), sigmoid colon (>75 years), splenic flexure (>80 years), stomach (>75 years), transverse colon (>75 years), urinary bladder (>80 years).

*The effect of sex on survival: balancing the factors reduces the gap, but does not eliminate it*

After the matching it was still present an association between sex and age clusters (Pearson’s χ2: 329.02; p<0.0005; Cramer’s V_(1)_: 0.027), and stage (Pearson’s χ2: 188.40; p<0.0005; Cramer’s V_(1)_: 0.021).

There still was some association between sex and histology per cancer site in the esophagus (Pearson’s χ2:42.74; Cramer’s V_(1)_:0.151; p<0.0005), kidney and renal pelvis (Pearson’s χ2:417.57; Cramer’s V_(1)_:0.118; p<0.0005), liver (Pearson’s χ2:54.18; Cramer’s V_(1)_:0.083; p<0.0005), lung (Pearson’s χ2:2,382.13; Cramer’s V_(1)_:0.107; p<0.0005), main bronchus (Pearson’s χ2:63.27; Cramer’s V_(1)_:0.163; p<0.0005), rectum (Pearson’s χ2:135.56; Cramer’s V_(1)_:0.091; p<0.0005), stomach (Pearson’s χ2:125.04; Cramer’s V_(1)_:0.125; p<0.0005), and urinary bladder (Pearson’s χ2:64.98; Cramer’s V_(1)_:0.080; p<0.0005). Thus, some unbalance was still present.

# Supplementary Figures and Tables

## Supplementary Figures


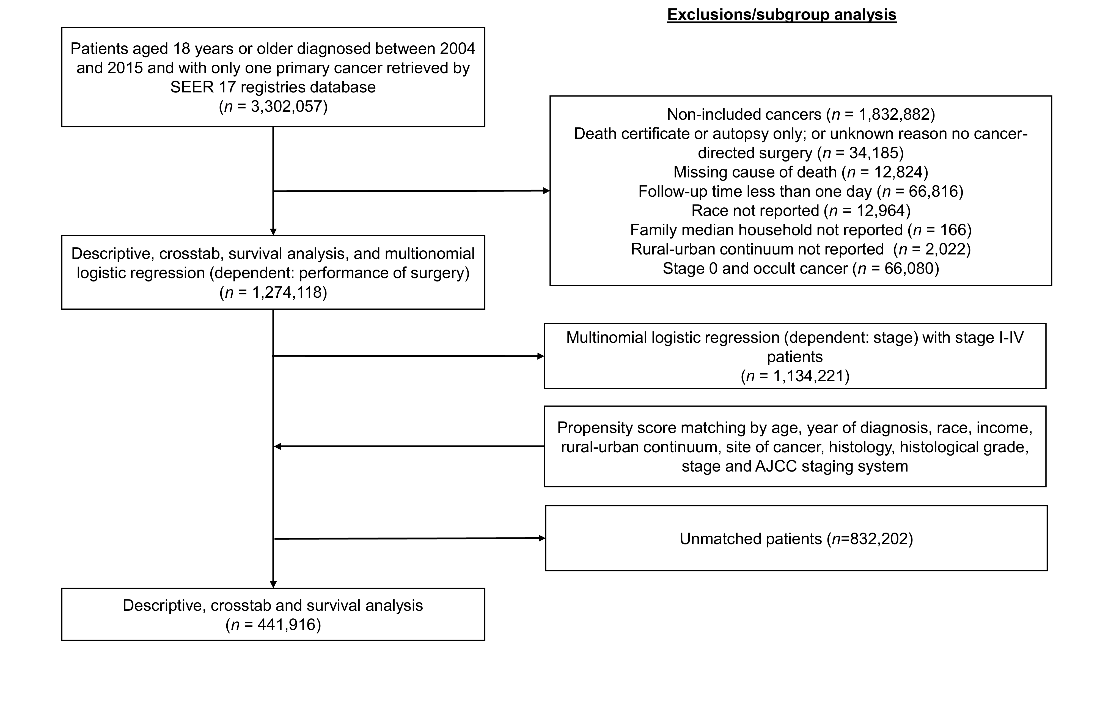


**Supplementary Figure 1. Flowchart of patients included and excluded in each statistical analysis.**


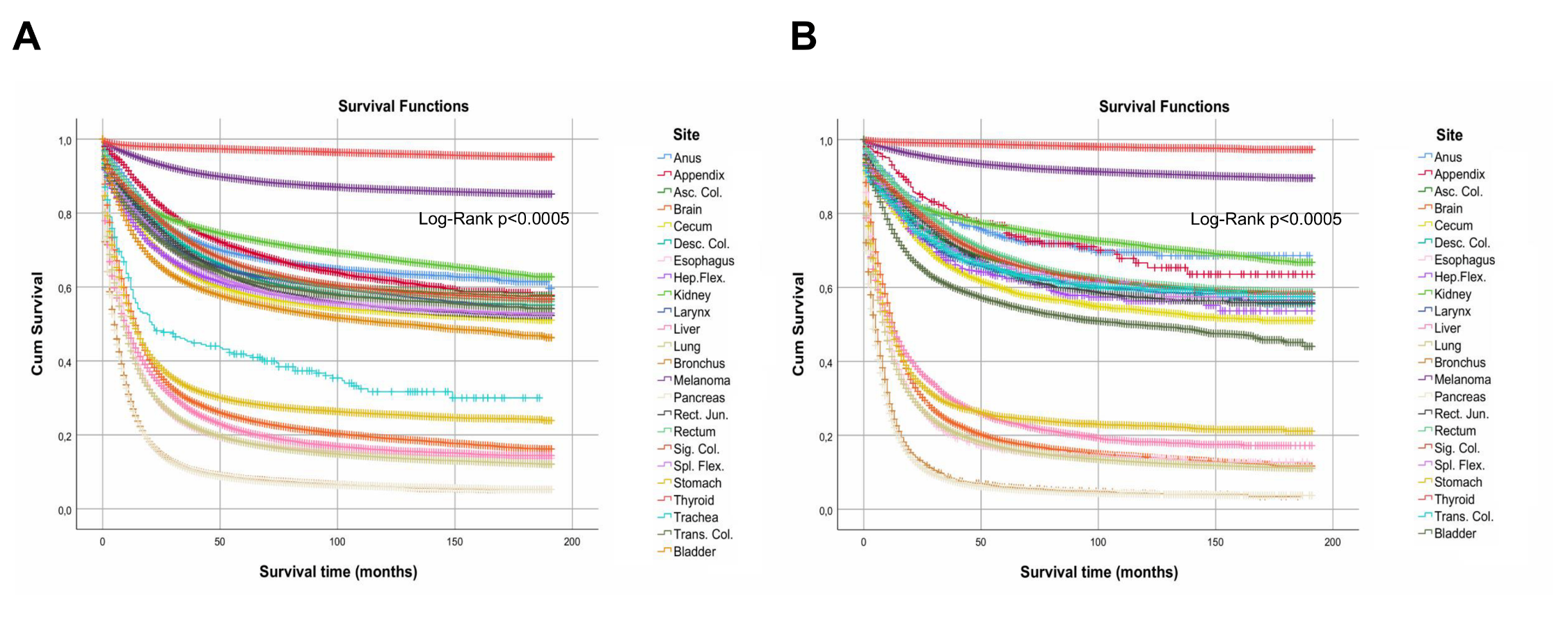


**Supplementary Figure 2. Kaplan-Meier plot of cancer specific survival by site of cancer.** (A) Before (Log-Rank χ^2^: 534,563.04; p<0.0005) (*n*=1,274,118) and (B) after propensity score matching (Log-Rank χ^2^: 198,822.06; p<0.0005) (*n*=441,916).


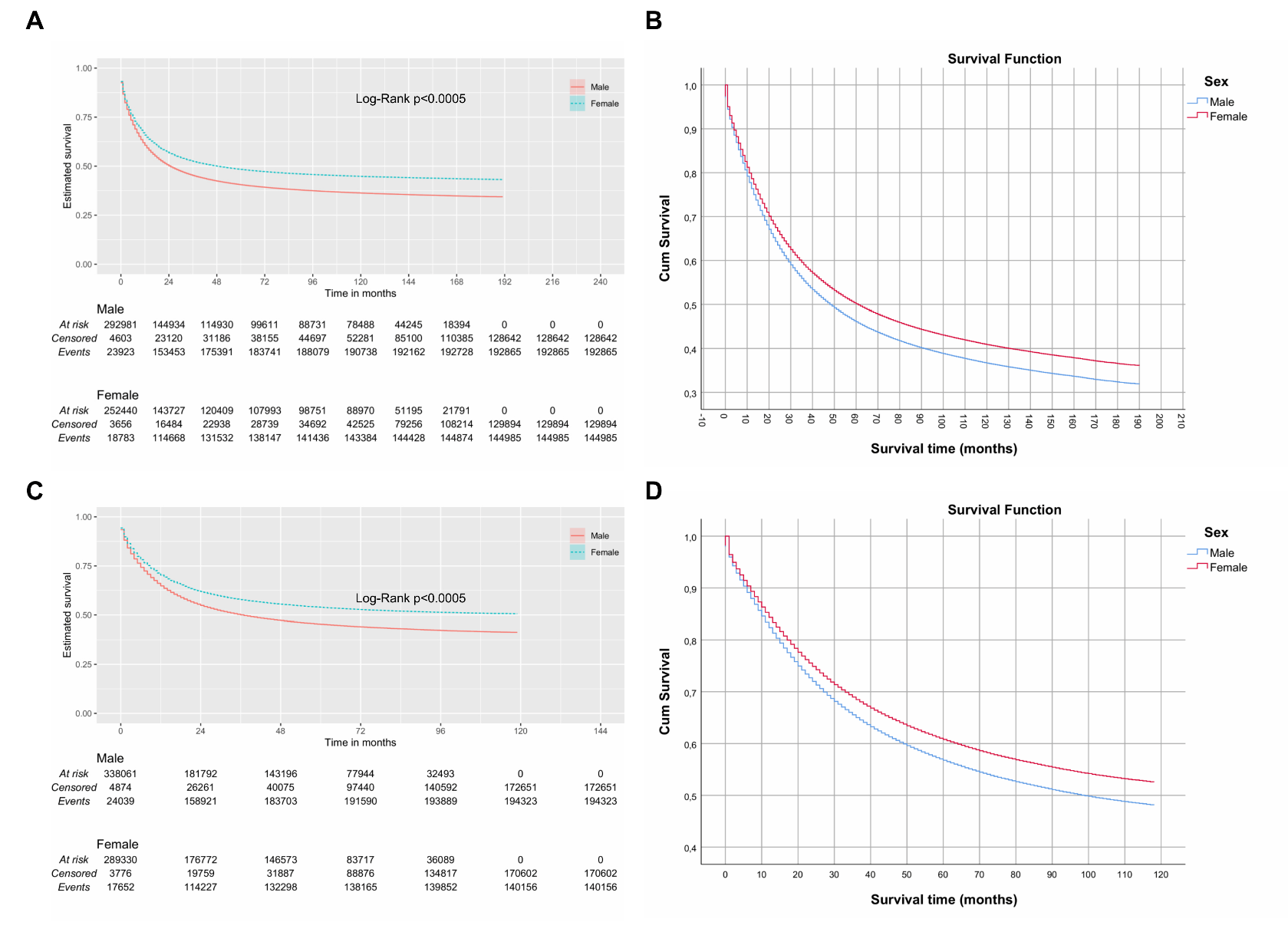


**Supplementary Figure 3. Kaplan-Meier and adjusted plots of cancer specific survival by** **period before matching.** (A) Periods of 2004-2009 and (B) 2010-2015.


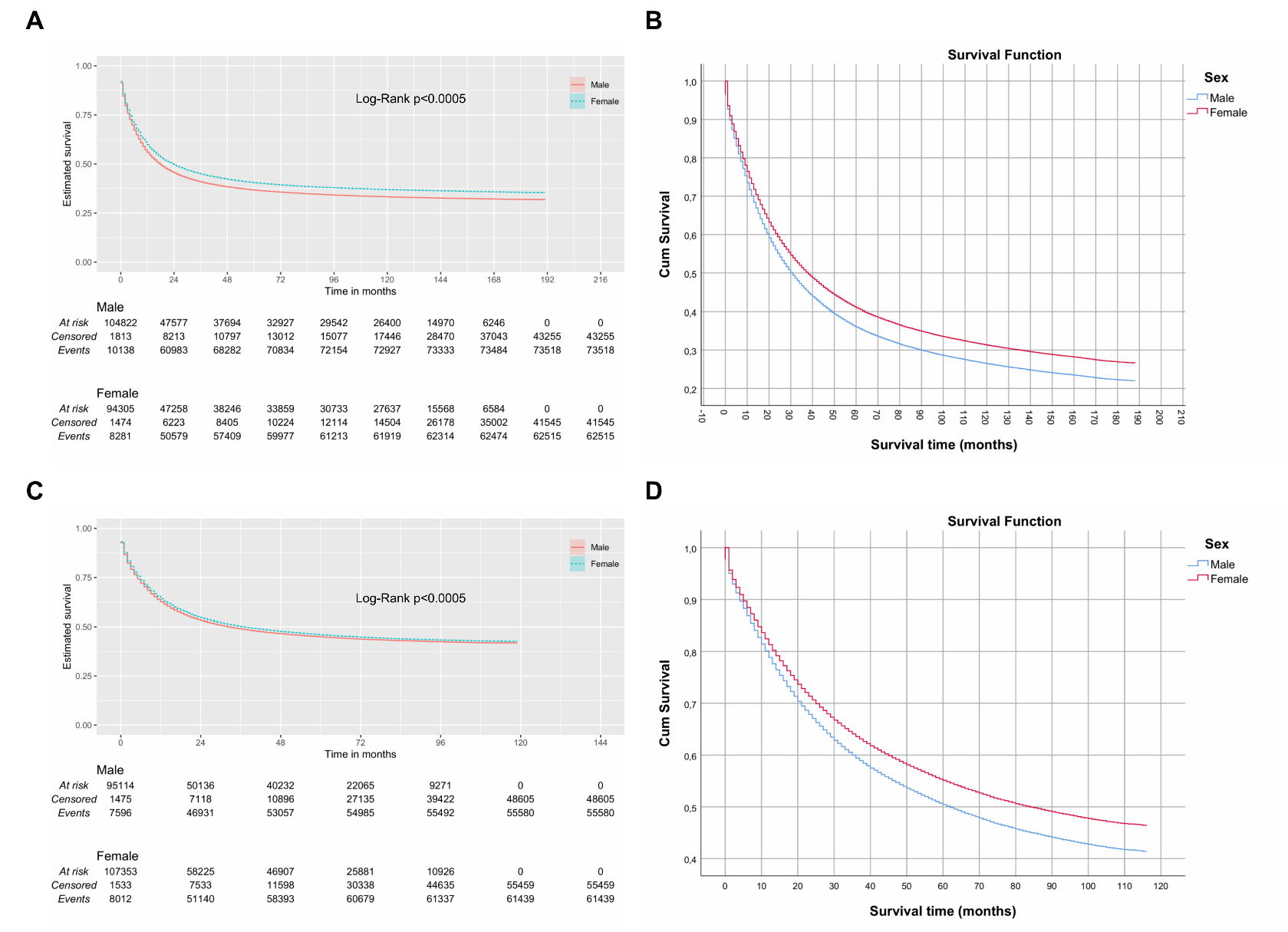


**Supplementary Figure 4. Kaplan-Meier and adjusted plots of cancer specific survival by** **period after matching.** (A) Periods of 2004-2009 and (B) 2010-2015.

## Supplementary Tables

**Supplementary Table 1. Classification of histology according to its frequency in each site for survival analysis.**

| **Site** | **First** | **Second** | **Third** | **Fourth** | **Fifth** | **Sixth** | **Seventh** |
| --- | --- | --- | --- | --- | --- | --- | --- |
| Anus, Anal Canal and Anorectum | 8070/3 | 8083/3 | 8071/3 | 8140/3 | 8124/3 | 8072/3 | 8720/3 |
| Appendix | 8480/3 | 8240/3 | 8140/3 | 8243/3 | 8490/3 | 8246/3 | 8244/3 |
| Ascending Colon | 8140/3 | 8480/3 | 8263/3 | 8210/3 | 8261/3 | 8481/3 | 8490/3 |
| Brain | 9440/3 | 9401/3 | 9400/3 | 9380/3 | 9450/3 | 9382/3 | 8000/3 |
| Cecum | 8140/3 | 8480/3 | 8263/3 | 8210/3 | 8261/3 | 8481/3 | 8490/3 |
| Descending Colon | 8140/3 | 8210/3 | 8263/3 | 8480/3 | 8261/3 | 8481/3 | 8010/3 |
| Esophagus | 8140/3 | 8070/3 | 8010/3 | 8490/3 | 8071/3 | 8000/3 | 8480/3 |
| Hepatic Flexure | 8140/3 | 8480/3 | 8263/3 | 8210/3 | 8261/3 | 8490/3 | 8481/3 |
| Kidney and Renal Pelvis | 8310/3 | 8312/3 | 8260/3 | 8317/3 | 8120/3 | 8130/3 | 8255/3 |
| Larynx | 8070/3 | 8071/3 | 8072/3 | 8010/3 | 8083/3 | 8051/3 | 8074/3 |
| Liver | 8170/3 | 8160/3 | 8000/3 | 8140/3 | 8010/3 | 8180/3 | 8174/3 |
| Lung | 8140/3 | 8041/3 | 8046/3 | 8070/3 | 8000/3 | 8012/3 | 8250/3 |
| Main Bronchus | 8041/3 | 8070/3 | 8140/3 | 8046/3 | 8010/3 | 8000/3 | 8246/3 |
| Melanoma of the Skin | 8720/3 | 8743/3 | 8742/3 | 8721/3 | 8772/3 | 8745/3 | 8744/3 |
| Pancreas | 8140/3 | 8010/3 | 8500/3 | 8000/3 | 8246/3 | 8480/3 | 8240/3 |
| Rectosigmoid Junction | 8140/3 | 8263/3 | 8210/3 | 8480/3 | 8261/3 | 8240/3 | 8010/3 |
| Rectum | 8140/3 | 8240/3 | 8263/3 | 8210/3 | 8480/3 | 8261/3 | 8070/3 |
| Sigmoid Colon | 8140/3 | 8210/3 | 8263/3 | 8480/3 | 8261/3 | 8010/3 | 8240/3 |
| Splenic Flexure | 8140/3 | 8480/3 | 8263/3 | 8210/3 | 8261/3 | 8010/3 | 8481/3 |
| Stomach | 8140/3 | 8490/3 | 8144/3 | 8936/3 | 8145/3 | 8240/3 | 8010/3 |
| Thyroid | 8260/3 | 8340/3 | 8341/3 | 8500/3 | 8330/3 | 8335/3 | 8510/3 |
| Trachea | 8070/3 | 8200/3 |  |  |  |  |  |
| Transverse Colon | 8140/3 | 8480/3 | 8210/3 | 8263/3 | 8261/3 | 8481/3 | 8000/3 |
| Urinary Bladder | 8130/3 | 8120/3 | 8070/3 | 8010/3 | 8000/3 | 8041/3 | 8140/3 |

**Supplementary Table 2. Classification of histology according to its frequency in each site for matching.**

| **Site** | **Most Common** | **Less common** |
| --- | --- | --- |
| Anus, Anal Canal and Anorectum | 8070/3 | 80071/3, 8083/3, 8140/3 |
| Appendix | 8140/3, 8240/3, 8480/3 | 8243/3, 8244/3, 8245/3, 8246/3, 8490/3 |
| Ascending Colon | 8140/3 | 8210/3, 8263/3, 8480/3 |
| Brain | 9440/3 | 8000/3, 9380/3, 9382/3, 9400/3, 9401/3, 9450/3 |
| Cecum | 8140/3 | 8210/3, 8261/3, 8263/3, 8480/3 |
| Descending Colon | 8140/3 | 8210/3, 8263/3, 8480/3 |
| Esophagus | 8140/3 | 8070/3 |
| Hepatic Flexure | 8140/3 | 8210/3, 8263/3, 8480/3 |
| Kidney and Renal Pelvis | 8310/3, 8312/3 | 8260/3, 8317/3, |
| Larynx | 8070/3 | 8071/3 |
| Liver | 8170/3 | 8000/3, 8010/3, 8140/3, 8160/3 |
| Lung | 8140/3 | 8041/3, 8046/3, 8070/3 |
| Main Bronchus | 8041/3, 8070/3 | 8000/3, 8010/3, 8046/3, 8140/3 |
| Melanoma of the Skin | 8720/3, 8743/3 | 8721/3, 8742/3 |
| Pancreas | 8140/3 | 8000/3, 8010/3, 8500/3 |
| Rectosigmoid Junction | 8140/3 | 8210/3, 8263/3 |
| Rectum | 8140/3 | 8240/3, 8263/3 |
| Sigmoid Colon | 8140/3 | 8210/3, 8263/3 |
| Splenic Flexure | 8140/3 | 8210/3, 8263/3, 8480/3 |
| Stomach | 8140/3 | 8144/3, 8145/3, 8490/3, 8936/3 |
| Thyroid | 8260/3 | 8340/3 |
| Trachea | 8070/3 | 8200/3 |
| Transverse Colon | 8140/3 | 8210/3, 8263/3, 8480/3 |
| Urinary Bladder | 8120/3, 8130/3 | 8000/3, 8010/3, 8070/3 |

**Supplementary Table 3. Frequencies of cancer stage by site (*n*=1,274,118).**

| **Site/Stage** | **I** | **II** | **III** | **IV** | **NA** |
| --- | --- | --- | --- | --- | --- |
| Anus, Anal Canal and Anorectum | 1,717 (14.9%) | 3,142 (27.2%) | 2,950 (25.6%) | 906 (7.8%) | 2,830 (24.5%) |
| Appendix | 1,471 (23.9%) | 1,650 (26.8%) | 603 (9.8%) | 1,814 (29.4%) | 628 (10.2%) |
| Ascending Colon | 7,336 (21.3%) | 10,077 (29.2%) | 9,082 (26.3%) | 6,624 (19.2%) | 1,353 (3.9%) |
| Brain | 0 | 0 | 0 | 0 | 41,187 (100%) |
| Cecum | 7,751 (18.8%) | 10,331 (25.1%) | 11,751 (28.6%) | 9,753 (23.7%) | 1,570 (3.8%) |
| Descending Colon | 2,187 (20.3%) | 2,863 (26.5%) | 2,945 (27.3%) | 2,328 (21.6%) | 466 (4.3%) |
| Esophagus | 4,385 (14.8%) | 4,514 (15.2%) | 6,482 (21.8%) | 10,283 (34.6%) | 4,040 (13.6%) |
| Hepatic Flexure | 1,568 (18.1%) | 2,603 (30.0%) | 2,189 (25.3%) | 1,886 (21.8%) | 419 (4.8%) |
| Kidney and Renal Pelvis | 49,415 (52.9%) | 8,037 (8.6%) | 13,313 (14.3%) | 17,985 (19.3%) | 4.674 (5.0%) |
| Larynx | 6,310 (32.3%) | 2,970 (15.2%) | 3,173 (16.3%) | 5,751 (29.5%) | 1,313 (6.7%) |
| Liver | 16,037 (29.5%) | 8,424 (15.5%) | 9,445 (17.4%) | 9,160 (16.9%) | 11,225 (20.7%) |
| Lung | 53,096 (15.3%) | 19,139 (5.5%) | 75,270 (21.7%) | 177,554 (51.2%) | 21,841 (6.3%) |
| Main Bronchus | 728 (3.7%) | 664 (3.4%) | 5,599 (28.3%) | 11,790 (59.5%) | 1,034 (5.2%) |
| Melanoma of the Skin | 81,710 (69.5%) | 13,122 (11.2%) | 8,493 (7.2%) | 5,224 (4.4%) | 9,051 (7.7%) |
| Pancreas | 6,618 (7.7%) | 19,694 (22.9%) | 7,195 (8.3%) | 43,903 (50.9%) | 8,777 (10.2%) |
| Rectosigmoid Junction | 4,343 (20.5%) | 4,485 (21.2%) | 6,128 (28.9%) | 5,171 (24.4%) | 1,058 (5.0%) |
| Rectum | 15,830 (25.9%) | 10,734 (17.6%) | 15,035 (24.6%) | 10,768 (17.6%) | 8,756 (14.3%) |
| Sigmoid Colon | 13,664 (25.8%) | 11,419 (21.6%) | 13,566 (25.6%) | 11,901 (22.5%) | 2,377 (4.5%) |
| Splenic Flexure | 856 (14.6%) | 1,757 (29.9%) | 1,696 (28.9%) | 1,375 (23.4%) | 186 (3.2%) |
| Stomach | 9,923 (19.7%) | 5,712 (11.3%) | 7,348 (14.6%) | 19,135 (37.9%) | 8,322 (16.5%) |
| Thyroid | 65,209 (69.9%) | 6,791 (7.3%) | 10,421 (11.2%) | 7,073 (7.6%) | 3,842 (4.1%) |
| Trachea | 0 | 0 | 0 | 0 | 272 (100%) |
| Transverse Colon | 3,037 (19.2%) | 4,913 (29.5%) | 3,991 (25.2%) | 3,322 (21.0%) | 581 (3.7%) |
| Urinary Bladder | 23,058 (44.6%) | 11,978 (23.2%) | 4,123 (8.0%) | 8,449 (16.3%) | 4,095 (7.9%) |

NA: not applied/not assigned

**Supplementary Table 4. Number of patients, events, and percentage of censoring by site of cancer before and after matching.**

|  | **Before PSM (*n=*1,274,118)** | | | **After PSM (*n*=441,916)** | | |
| --- | --- | --- | --- | --- | --- | --- |
| **Site** | **Total *n*** | **Events** | **Censored (%)** | **Total *n*** | **Events** | **Censored (%)** |
| Anus, Anal Canal and Anorectum | 11,545 | 3,767 | 67.4 | 480 | 131 | 72.7 |
| Appendix | 6,166 | 2,037 | 67.0 | 310 | 85 | 72.6 |
| Ascending Colon | 34,472 | 12,455 | 63.9 | 6,830 | 2,319 | 66.0 |
| Brain | 41,187 | 31,200 | 24.2 | 12,078 | 9,789 | 19.0 |
| Cecum | 41,156 | 17,239 | 58.1 | 7,226 | 2,940 | 59.3 |
| Descending Colon | 10,789 | 4,115 | 6,674 | 1,134 | 416 | 63.3 |
| Esophagus | 29,704 | 23,322 | 21.5 | 1,882 | 1,499 | 20.4 |
| Hepatic Flexure | 8,665 | 3,522 | 59.4 | 554 | 208 | 62.5 |
| Kidney and Renal Pelvis | 93,424 | 27,216 | 70.9 | 30,160 | 7,868 | 73.9 |
| Larynx | 19,517 | 7,176 | 63.2 | 2,102 | 755 | 64.1 |
| Liver | 54,291 | 40,493 | 25.4 | 7,824 | 5,609 | 28.3 |
| Lung | 346,900 | 271,804 | 21.6 | 208,800 | 166,525 | 20.2 |
| Main Bronchus | 19,815 | 17,295 | 12.7 | 2,386 | 2,144 | 11.4 |
| Melanoma of the Skin | 117,600 | 14,341 | 87.8 | 53,178 | 4,401 | 91.7 |
| Pancreas | 86,187 | 76,744 | 11.0 | 25,968 | 23,659 | 8.9 |
| Rectosigmoid Junction | 21.185 | 86,82 | 59.0 | 3,098 | 1,190 | 61.6 |
| Rectum | 61,123 | 22,702 | 62.9 | 16,484 | 5,768 | 65.0 |
| Sigmoid Colon | 52,927 | 19,340 | 63.5 | 18,450 | 6,551 | 64.5 |
| Splenic Flexure | 5,870 | 2,382 | 59.4 | 316 | 114 | 63.9 |
| Stomach | 50,440 | 34,517 | 31.6 | 8,014 | 5,747 | 28.3 |
| Thyroid | 93,336 | 3,313 | 96.5 | 23,040 | 436 | 98.1 |
| Trachea | 272 | 167 | 38.6 |  |  |  |
| Transverse Colon | 15,844 | 60,68 | 61.7 | 1,476 | 519 | 64.8 |
| Urinary Bladder | 51,703 | 22,432 | 56.6 | 10,126 | 4,409 | 56.5 |

**Supplementary Table 5. Log-Rank test of cancer specific survival by sex according to site of cancer before and after matching.**

|  | **Before PSM (*n=*1,274,118)** | | **After PSM (*n*=441,916)** | | |
| --- | --- | --- | --- | --- | --- |
| **Site** | **χ^2^** | ***p*-value** | **χ^2^** | ***p*-value** |  |
| Anus, Anal Canal and Anorectum | 107.85 | <0.0005 | 12.43 | <0.0005 |  |
| Appendix | 1.27 | 0.259 | 0.799 | 0.371 |  |
| Ascending Colon | 4.37 | 0.037 | 6.92 | 0.009 |  |
| Brain | 2.53 | 0.112 | 15.28 | <0.0005 |  |
| Cecum | 4.82 | 0.028 | 5.35 | 0.021 |  |
| Descending Colon | 0.494 | 0.482 | 0.31 | 0.861 |  |
| Esophagus | 2.95 | 0.086 | 0.10 | 0.750 |  |
| Hepatic Flexure | 1.89 | 0.169 | 0.2 | 0.875 |  |
| Kidney and Renal Pelvis | 123.62 | <0.0005 | 8.69 | 0.003 |  |
| Larynx | 13.47 | <0.0005 | 5.67 | 0.017 |  |
| Liver | 30.70 | <0.0005 | 23.11 | <0.0005 |  |
| Lung | 2,797.42 | <0.0005 | 965.71 | <0.0005 |  |
| Main Bronchus | 20.15 | <0.0005 | 1.87 | 0.172 |  |
| Melanoma of the Skin | 1,164.22 | <0.0005 | 149.72 | <0.0005 |  |
| Pancreas | 1.42 | 0.233 | 16.18 | <0.0005 |  |
| Rectosigmoid Junction | 12.93 | <0.0005 | 4.18 | 0.041 |  |
| Rectum | 47.08 | <0.0005 | 48.70 | <0.0005 |  |
| Sigmoid Colon | 7.26 | 0.007 | 11.37 | 0.001 |  |
| Splenic Flexure | 0.15 | 0.699 | 0.45 | 0.831 |  |
| Stomach | 143.67 | <0.0005 | 9.30 | 0.002 |  |
| Thyroid | 922.55 | <0.0005 | 22.14 | <0.0005 |  |
| Trachea | 0.31 | 0.580 |  |  |  |
| Transverse Colon | 1.17 | 0.279 | 0.40 | 0.844 |  |
| Urinary Bladder | 609.08 | <0.0005 | 12.06 | 0.001 |  |

**Supplementary Table 6. One, 3-, 5-, 10- and 15-year survival before matching (n=1,274,118)**

| **Year** | **Sex** | **Number at risk** | **Number of events** | **Survival (95% CI)** |
| --- | --- | --- | --- | --- |
| 1 | Female | 384,459 | 181,369 | 68.2% (68.1% - 68.4%) |
|  | Male | 413,660 | 248,119 | 62.9% (62.7% - 63.0%) |
|  |  |  |  |  |
| 3 | Female | 291,969 | 69,990 | 55.3% (55.2% - 55.5%) |
|  | Male | 289,427 | 94,252 | 44.9% (44.8% - 45.0%) |
|  |  |  |  |  |
| 5 | Female | 229,117 | 20,191 | 51.3% (51.2% - 51.4%) |
|  | Male | 216,922 | 26,798 | 43.1% (43.0% - 43.2%) |
|  |  |  |  |  |
| 10 | Female | 90,633 | 11,990 | 47.7% (47.6% - 47.8%) |
|  | Male | 80,070 | 15,892 | 38.8% (38.6% - 38.9%) |
|  |  |  |  |  |
| 15 | Female | 10,487 | 1,578 | 46.2% (46.0% - 46.3%) |
|  | Male | 8,814 | 2,105 | 36.9% (36.8% - 37.1%) |

**Supplementary Table 7. One, 3-, 5-, 10- and 15-year survival after matching (n=441,916)**

| **Year** | **Sex** | **Number at risk** | **Number of events** | **Survival (95% CI)** |
| --- | --- | --- | --- | --- |
| 1 | Female | 132,184 | 81,473 | 62.1% (61.9% - 62.3%) |
|  | Male | 124,606 | 87,826 | 59.0% (58.8% - 59.2%) |
|  |  |  |  |  |
| 3 | Female | 94,295 | 29,379 | 47.7% (47.5% - 47.9%) |
|  | Male | 86,890 | 28,820 | 44.7% (44.4% - 44.9%) |
|  |  |  |  |  |
| 5 | Female | 72,054 | 8,021 | 43.4% (43.2% - 43.6%) |
|  | Male | 66,115 | 7,462 | 40.6% (40.4% - 40.8%) |
|  |  |  |  |  |
| 10 | Female | 28,165 | 4,485 | 39.8% (39.6% - 40.0%) |
|  | Male | 26,911 | 4,399 | 37.0% (36.8% - 37.2%) |
|  |  |  |  |  |
| 15 | Female | 3,058 | 591 | 38.2% (38.0% - 38.5%) |
|  | Male | 2,954 | 586 | 35.6% (35.3% - 35.8%) |

**Supplementary Table 8. Hazard of cancer specific death of female sex compared to male sex before propensity score matching (n= 1,274,118)**

|  | **Univariable** | | **Multivariable** | |
| --- | --- | --- | --- | --- |
| **Subgroup** | **HR (95%CI)** | ***p*-value** | **HR (95%CI)** | ***p*-value** |
| Entire cohort | 0.801 (0.797 – 0.805) | <0.0005 | 0.876 (0.866 – 0.886) | <0.0005 |
| Anus, Anal Canal and Anorectum | 0.713 (0.668 – 0.760) | <0.0005 | 0738 (0.667 – 0.818) | <0.0005 |
| Appendix | 0.951 (0.872 – 1.038) | 0.261 | 0.872 (0.765 – 0.994) | 0.040 |
| Ascending Colon | 0.963 (0.930 – 0.998) | 0.038 | 0.893 (0.839 – 0.950) | <0.0005 |
| Brain | 0.982 (0.961 – 1.005) | 0.119 | 0.874 (0.845 – 0.904) | <0.0005 |
| Cecum | 0.967 (0.939 – 0.997) | 0.029 | 0.850 (0.807 – 0.897) | <0.0005 |
| Descending Colon | 1.022 (0.961 – 1.087) | 0.485 | 0.989 (0.912 – 1.073) | 0.791 |
| Esophagus | 1.027 (0.995 – 1.061) | 0.094 | 0.935 (0.870 – 1.005) | 0.069 |
| Hepatic Flexure | 0.955 (0.894 – 1.020) | 0.172 | 0.931 (0.827 – 1.049) | 0.240 |
| Kidney and Renal Pelvis | 0.869 (0.848 – 0.891) | <0.0005 | 0.917 (0.880 – 0.954) | <0.0005 |
| Larynx | 1.113 (1.051 – 1.179) | <0.0005 | 0.976 (0.786 – 1.213) | 0.830 |
| Liver | 0.939 (0.918 – 0.961) | <0.0005 | 0.811 (0.774 – 0.850) | <0.0005 |
| Lung | 0.821 (0.815 – 0.827) | <0.0005 | 0.844 (0.832 – 0.855) | <0.0005 |
| Main Bronchus | 0.937 (0.909 – 0.965) | <0.0005 | 0.900 (0.859 – 0.943) | <0.0005 |
| Melanoma of the Skin | 0.550 (0.531 – 0.569) | <0.0005 | 0.698 (0.668 – 0.729) | <0.0005 |
| Pancreas | 0.992 (0.978 – 1.006) | 0.257 | 0.903 (0.868 – 0.939) | <0.0005 |
| Rectosigmoid Junction | 0.925 (0.887 – 0.966) | <0.0005 | 0.891 (0.823 – 0.964) | 0.004 |
| Rectum | 0.912 (0.888 – 0.936) | <0.0005 | 0.869 (0.832 – 0.908) | <0.0005 |
| Sigmoid Colon | 0.962 (0.935 – 0.990) | 0.007 | 0.890 (0.843 – 0.938) | <0.0005 |
| Splenic Flexure | 1.016 (0.937 – 1.101) | 0.701 | 0.820 (0.704 – 0.955) | 0.011 |
| Stomach | 0.878 (0.859 – 0.897) | <0.0005 | 0.926 (0.893 – 0.959) | <0.0005 |
| Thyroid | 0.361 (0.337 – 0.387) | <0.0005 | 0.619 (0.542 – 0.708) | <0.0005 |
| Trachea | 0.917 (0.671 – 1.253) | 0.586 | 0.966 (0.662 – 1.409) | 0.854^*^ |
| Transverse Colon | 1.028 (0.977 – 1.081) | 0.283 | 0.913 (0.826 – 1.004) | 0.076 |
| Urinary Bladder | 1.416 (1.377 – 1.456) | <0.0005 | 1.172 (1.106 – 1.241) | <0.0005 |

*Bootstrap p-value.

**Supplementary Table 9. Hazard of cancer specific death of female sex compared to male sex after propensity score matching (n=441,916).**

|  | **Univariable** | | **Multivariable** | |
| --- | --- | --- | --- | --- |
| **Subgroup** | **HR (95%CI)** | ***p*-value** | **HR (95%CI)** | ***p*-value** |
| Entire cohort | 0.922 (0.915 – 0.929) | <0.0005 | 0.888 (0.864 – 0.912) | <0.0005 |
| Anus, Anal Canal and Anorectum | 0.537 (0.377 – 0.764) | 0.001 | 0.519 (0.344 – 0.783) | 0.003^*^ |
| Appendix | 0.823 (0.537 – 1.263) | 0.373 | 0.840 (0.537 – 1.313) | 0.476^#^ |
| Ascending Colon | 0.897 (0.827 – 0.973) | 0.009 | 0.917 (0.844 – 0.996) | 0.039 |
| Brain | 0.926 (0.890 – 0.963) | <0.0005 | 0.943 (0.906 – 0.981) | 0.004^1^ |
| Cecum | 0.919 (0.855 – 0.988) | 0.022 | 0.910 (0.846 – 0.979) | 0.011 |
| Descending Colon | 1.017 (0.839 – 1.233) | 0.862 | 1.033 (0.852 – 1.252) | 0.758^#^ |
| Esophagus | 0.984 (0.889 – 1.089) | 0.757 | 1.006 (0.908 – 1.115) | 0.893^#^ |
| Hepatic Flexure | 0.978 (0.745 – 1.284) | 0.875 | 0.976 (0.743 – 1.283) | 0.888^#^ |
| Kidney and Renal Pelvis | 0.936 (0.895 – 0.978) | 0.003 | 0.931 (0.890 – 0.974) | 0.002^2^ |
| Larynx | 1.188 (1.030 – 1.371) | 0.018 | 1.180 (1.023 – 1.362) | 0.025^#^ |
| Liver | 0.883 (0.838 – 0.930) | <0.0005 | 0.651 (0.448 – 0.945) | 0.024 |
| Lung | 0.863 (0.855 – 0.871) | <0.0005 | 0.872 (0.827 – 0.920) | <0.0005 |
| Main Bronchus | 0.945 (0.868 – 1.029) | 0.195 | 0.927 (0.850 – 1.012) | 0.090 |
| Melanoma of the Skin | 0.689 (0.649 – 0.732) | <0.0005 | 0.734 (0.691 – 0.780) | <0.0005 |
| Pancreas | 0.952 (0.928 – 0.976) | <0.0005 | 0.929 (0.906 – 0.953) | <0.0005^3^ |
| Rectosigmoid Junction | 0.889 (0.793 – 0.996) | 0.042 | 0.832 (0.741 – 0.935) | 0.002^4^ |
| Rectum | 0.832 (0.790 – 0.877) | <0.0005 | 0.863 (0.819 – 0.910) | <0.0005 |
| Sigmoid Colon | 0.920 (0.877 – 0.966) | 0.001 | 0.926 (0.882 – 0.973) | 0.002 |
| Splenic Flexure | 0.961 (0.666 – 1.388) | 0.832 | 0.916 (0.629 – 1.336) | 0.650^#^ |
| Stomach | 0.925 (0.878 – 0.974) | 0.003 | 0.945 (0.896 – 0.995) | 0.033^7^ |
| Thyroid | 0.632 (0.521 – 0.767) | <0.0005 | 0.629 (0.515 – 0.769) | <0.0005 |
| Transverse Colon | 0.983 (0.828 – 1.168) | 0.845 | 0.972 (0.818 – 1.155) | 0.750^#^ |
| Urinary Bladder | 1.109 (1.046 – 1.177) | 0.001 | 1.036 (0.975 – 1.100) | 0.253^8^ |

^*^Bootstrap p-value. Model with all variables.

^#*^Bootstrap p-value. Model with propensity score and histologies.

^1^In the presence of the three-way interaction (p=0.069) HR: 0.852 (0.772 – 0.940), p=0.001

^2^In the presence of the three-way interaction (p=0.159) HR: 0.966 (0.887 – 1.053), p=0.437

^3^In the presence of the three-way interaction (p=0.013), HR: 0.993 (0.906 – 1.090), p=0.889

^4^In the presence of the three-way interaction (p=0.030), HR: 0.492 (0.107 – 2.253), p=0.361

^5^In the presence of the three-way interaction (p=0.024), HR: 0.777 (0.690 – 0.876), p<0.0005

^6^In the presence of the three-way interaction (p=0.002), HR: 0.868 (0.768 – 0.981), p=0.023

^7^In the presence of the three-way interaction (p=0.684), HR: 0.843 (0.761 – 0.933), p=0.001

^8^In the presence of the three-way interaction (p=0.683), HR: 1.425 (1.014 – 2.003), p=0.042

**Supplementary Table 10. Hazard of cancer specific death of years compared to 2004, by sex, before propensity score matching (n=1,274,118)**

|  | **Female** | | | | **Male** | | | |
| --- | --- | --- | --- | --- | --- | --- | --- | --- |
|  | **Univariable** | | **Multivariable^*^** | | **Univariable** | | **Multivariable^*^** | |
| **Year** | **HR (95%CI)** | ***p*-value** | **HR (95%CI)** | ***p*-value** | **HR (95%CI)** | ***p*-value** | **HR (95%CI)** | ***p*-value** |
| 2005 | 0.955 (0.938 – 0.972) | <0.0005 | 0.976 (0.954 – 0.994) | 0.008 | 0.971 (0.956 – 0.986) | <0.0005 | 0.984 (0.969 – 1.000) | 0.049 |
| 2006 | 0.936 (0.920 – 0.953) | <0.0005 | 0.942 (0.925 – 0.959) | <0.0005 | 0.945 (0.930 – 0.960) | <0.0005 | 0.946 (0.931 – 0.961) | <0.0005 |
| 2007 | 0.907 (0.891 – 0.923) | <0.0005 | 0.922 (0.906 – 0.939) | <0.0005 | 0.920 (0.905 – 0.934) | <0.0005 | 0.928 (0.914 – 0.943) | <0.0005 |
| 2008 | 0.878 (0.862 – 0.893) | <0.0005 | 0.911 (0.895 – 0.927) | <0.0005 | 0.887 (0.874 – 0.901) | <0.0005 | 0.893 (0.880 – 0.907) | <0.0005 |
| 2009 | 0.854 (0.839 – 0.869) | <0.0005 | 0.883 (0.867 – 0.899) | <0.0005 | 0.873 (0.860 – 0.887) | <0.0005 | 0.869 (0.856 – 0.883) | <0.0005 |
| 2010 | 0.833 (0.818 – 0.848) | <0.0005 | 0.853 (0.837 – 0.868) | <0.0005 | 0.880 (0.867 – 0.894) | <0.0005 | 0.855 (0.842 – 0.868) | <0.0005 |
| 2011 | 0.813 (0.799 – 0.828) | <0.0005 | 0.836 (0.821 – 0.851) | <0.0005 | 0.850 (0.837 – 0.863) | <0.0005 | 0.830 (0.817 – 0.843) | <0.0005 |
| 2012 | 0.788 (0.774 – 0.803) | <0.0005 | 0.818 (0.804 – 0.834) | <0.0005 | 0.834 (0.821 – 0.847) | <0.0005 | 0.819 (0.806 – 0.832) | <0.0005 |
| 2013 | 0.779 (0.765 – 0.794) | <0.0005 | 0.797 (0.783 – 0.812) | <0.0005 | 0.814 (0.801 – 0.827) | <0.0005 | 0.801 (0.789 – 0.814) | <0.0005 |
| 2014 | 0.754 (0.741 – 0.768) | <0.0005 | 0.782 (0.768 – 0.796) | <0.0005 | 0.778 (0.766 – 0.790) | <0.0005 | 0.770 (0.758 – 0.783) | <0.0005 |
| 2015 | 0.723 (0.710 – 0.736) | <0.0005 | 0.741 (0.727 – 0.755) | <0.0005 | 0.739 (0.728 – 0.751) | <0.0005 | 0.740 (0.728 – 0.752) | <0.0005 |

**Supplementary Table 11. Hazard of cancer specific death of years compared to 2004, by sex, after propensity score matching (n=441,916)**

|  | **Female** | | | | **Male** | | | |
| --- | --- | --- | --- | --- | --- | --- | --- | --- |
|  | **Univariable** | | **Multivariable** | | **Univariable** | | **Multivariable** | |
| **Year** | **HR (95%CI)** | ***p*-value** | **HR (95%CI)** | ***p*-value** | **HR (95%CI)** | ***p*-value** | **HR (95%CI)** | ***p*-value** |
| 2005 | 0.947 (0.921 – 0.973) | <0.0005 | 0.978 (0.952 – 1.006) | 0.119 | 0.962 (0.938 – 0.987) | 0.003 | 0.983 (0.963 – 1.009) | 0.193 |
| 2006 | 0.938 (0.913 – 0.964) | <0.0005 | 0.942 (0.916 – 0.968) | <0.0005 | 0.939 (0.916 – 0.963) | <0.0005 | 0.938 (0.913 – 0.963) | <0.0005 |
| 2007 | 0.903 (0.878 – 0.928) | <0.0005 | 0.919 (0.894 – 0.945) | <0.0005 | 0.912 (0.889 – 0.935) | <0.0005 | 0.920 (0.897 – 0.945) | <0.0005 |
| 2008 | 0.851 (0.828 – 0.875) | <0.0005 | 0.908 (0.883 – 0.933) | <0.0005 | 0.857 (0.836 – 0.879) | <0.0005 | 0.888 (0.865 – 0.912) | <0.0005 |
| 2009 | 0.838 (0.816 – 0.861) | <0.0005 | 0.886 (0.863 – 0.911) | <0.0005 | 0.861 (0.840 – 0.883) | <0.0005 | 0.876 (0.853 – 0.899) | <0.0005 |
| 2010 | 0.852 (0.829 – 0.875) | <0.0005 | 0.858 (0.834 – 0.882) | <0.0005 | 0.802 (0.781 – 0.824) | <0.0005 | 0.849 (0.826 – 0.873) | <0.0005 |
| 2011 | 0.828 (0.806 – 0.851) | <0.0005 | 0.832 (0.809 – 0.856) | <0.0005 | 0.781 (0.760 – 0.802) | <0.0005 | 0.839 (0.816 – 0.862) | <0.0005 |
| 2012 | 0.799 (0.778 – 0.821) | <0.0005 | 0.817 (0.794 – 0.840) | <0.0005 | 0.754 (0.734 – 0.774) | <0.0005 | 0.831 (0.808 – 0.855) | <0.0005 |
| 2013 | 0.785 (0.764 – 0.807) | <0.0005 | 0.795 (0.773 – 0.817) | <0.0005 | 0.749 (0.729 – 0.769) | <0.0005 | 0.822 (0.799 – 0.845) | <0.0005 |
| 2014 | 0.752 (0.732 – 0.773) | <0.0005 | 0.776 (0.755 – 0.798) | <0.0005 | 0.712 (0.693 – 0.732) | <0.0005 | 0.790 (0.768 – 0.812) | <0.0005 |
| 2015 | 0.705 (0.685 – 0.725) | <0.0005 | 0.727 (0.707 – 0.748) | <0.0005 | 0.663 (0.645 – 0.681) | <0.0005 | 0.770 (0.749 – 0.792) | <0.0005 |

**Supplementary Table 12. Hazard of cancer specific death of female compared to male by year before propensity score matching (n=1,274,118)**

|  | **Univariable** | | **Multivariable** | |
| --- | --- | --- | --- | --- |
| **Year** | **HR (95%CI)** | ***p*-value** | **HR (95%CI)** | ***p*-value** |
| 2004 | 0.820 (0.807 – 0.834) | <0.0005 | 0.891 (0.876 – 0.906) | <0.0005 |
| 2005 | 0.807 (0.794 – 0.821) | <0.0005 | 0.879 (0.864 – 0.894) | <0.0005 |
| 2006 | 0.812 (0.799 – 0.826) | <0.0005 | 0.881 (0.865 – 0.896) | <0.0005 |
| 2007 | 0.809 (0.796 – 0.823) | <0.0005 | 0.880 (0.865 – 0.896) | <0.0005 |
| 2008 | 0.811 (0.798 – 0.825) | <0.0005 | 0.899 (0.883 – 0.914) | <0.0005 |
| 2009 | 0.803 (0.790 – 0.816) | <0.0005 | 0.891 (0.876 – 0.907) | <0.0005 |
| 2004-2009 | 0.810 (0.805 – 0.816) | <0.0005 | 0.881 (0.867 – 0.895) | <0.0005 |
| 2010 | 0.778 (0.765 – 0.791) | <0.0005 | 0.878 (0.863 – 0.893) | <0.0005 |
| 2011 | 0.786 (0.773 – 0.800) | <0.0005 | 0.889 (0.874 – 0.905) | <0.0005 |
| 2012 | 0.779 (0.766 – 0.792) | <0.0005 | 0.885 (0.870 – 0.901) | <0.0005 |
| 2013 | 0.791 (0.778 – 0.804) | <0.0005 | 0.880 (0.865 – 0.896) | <0.0005 |
| 2014 | 0.801 (0.788 – 0.815) | <0.0005 | 0.894 (0.878 – 0.910) | <0.0005 |
| 2015 | 0.811 (0.797 – 0.825) | <0.0005 | 0.885 (0.869 – 0.901) | <0.0005 |
| 2010-2015 | 0.791 (0.786 – 0.797) | <0.0005 | 0.871 (0.857 – 0.886) | <0.0005 |

**Supplementary Table 13. Hazard of cancer specific death of female compared to male by year after propensity score matching (n=441,916)**

|  | **Univariable** | | **Multivariable** | |
| --- | --- | --- | --- | --- |
| **Year** | **HR (95%CI)** | ***p*-value** | **HR (95%CI)** | ***p*-value** |
| 2004 | 0.911 (0.887 – 0.935) | <0.0005 | 0.872 (0.849 – 0.896) | <0.0005 |
| 2005 | 0.898 (0.874 – 0.922) | <0.0005 | 0.868 (0.846 – 0.892) | <0.0005 |
| 2006 | 0.910 (0.886 – 0.934) | <0.0005 | 0.873 (0.850 – 0.896) | <0.0005 |
| 2007 | 0.903 (0.880 – 0.927) | <0.0005 | 0.869 (0.847 – 0.892) | <0.0005 |
| 2008 | 0.905 (0.882 – 0.928) | <0.0005 | 0.887 (0.864 – 0.911) | <0.0005 |
| 2009 | 0.887 (0.864 – 0.910) | <0.0005 | 0.896 (0.872 – 0.919) | <0.0005 |
| 2004-2009 | 0.901 (0.892 – 0.911) | <0.0005 | 0.885 (0.852 – 0.920) | <0.0005 |
| 2010 | 0.968 (0.941 – 0.995) | 0.023 | 0.884 (0.860 – 0.910) | <0.0005 |
| 2011 | 0.967 (0.941 – 0.995) | 0.020 | 0.872 (0.848 – 0.897) | 0.002 |
| 2012 | 0.967 (0.941 – 0.995) | 0.019 | 0.874 (0.849 – 0.899) | <0.0005 |
| 2013 | 0.956 (0.929 – 0.983) | 0.002 | 0.862 (0.838 – 0.887) | <0.0005 |
| 2014 | 0.963 (0.936 – 0.990) | 0.008 | 0.883 (0.858 – 0.908) | <0.0005 |
| 2015 | 0.968 (0.940 – 0.996) | 0.025 | 0.870 (0.845 – 0.896) | <0.0005 |
| 2010-2015 | 0.965 (0.954 – 0.976) | <0.0005 | 0.883 (0.848 – 0.919) | <0.0005 |

**Supplementary Table 14. One, 3-, 5-, 10- and 15-year survival before matching by** **period (n=1,274,118)**

| **Year** | **Time period** | **Sex** | **Number at risk** | **Number of events** | **Survival (95% CI)** |
| --- | --- | --- | --- | --- | --- |
| 1 | 2004-2009 | Female | 174,345 | 91,425 | 65.9% (65.7% - 66.0%) |
|  |  | Male | 186,048 | 122,690 | 60.6% (60.4% - 60.8%) |
|  |  |  |  |  |  |
|  | 2010-2015 | Female | 210,114 | 89,944 | 70.3% (70.2% - 70.5%) |
|  |  | Male | 227,612 | 125,429 | 64.8% (64.7% - 65.0%) |
|  |  |  |  |  |  |
| 3 | 2004-2009 | Female | 130,429 | 34,079 | 52.5% (52.3% - 52.7%) |
|  |  | Male | 127,778 | 44,875 | 45.2% (45.1% - 45.4%) |
|  |  |  |  |  |  |
|  | 2010-2015 | Female | 161,540 | 35,911 | 57.8% (57.7% - 58.0%) |
|  |  | Male | 161,649 | 49,377 | 50.0% (49.9% - 50.2%) |
|  |  |  |  |  |  |
| 5 | 2004-2009 | Female | 113,962 | 9,989 | 48.4% (48.2% - 48.5%) |
|  |  | Male | 107,008 | 12,810 | 40.5% (40.4% - 40.7%) |
|  |  |  |  |  |  |
|  | 2010-2015 | Female | 115,155 | 10,202 | 53.9% (53.7% - 54.1%) |
|  |  | Male | 109,914 | 13,988 | 45.4% (45.2% - 45.5%) |
|  |  |  |  |  |  |
| 10 | 2004-2009 | Female | 90,633 | 7,891 | 44.8% (44.6% - 45.0%) |
|  |  | Male | 80,070 | 10,363 | 36.3% (36.1% - 36.5%) |
|  |  |  |  |  |  |
|  | 2010-2015 | Female | --------- | --------- | --------- |
|  |  | Male | --------- | --------- | --------- |
|  |  |  |  |  |  |
| 15 | 2004-2009 | Female | 10,487 | 1,578 | 43.4% (43.2% - 43.6%) |
|  |  | Male | 8,814 | 2,105 | 34.6% (34.4% - 34.8%) |
|  |  |  |  |  |  |
|  | 2010-2015 | Female | --------- | --------- | --------- |
|  |  | Male | --------- | --------- | --------- |

**Supplementary Table 15. One, 3-, 5-, 10- and 15-year survival after matching by** **period (n=441,916)**

| **Year** | **Time period** | **Sex** | **Number at risk** | **Number of events** | **Survival (95% CI)** |
| --- | --- | --- | --- | --- | --- |
| 1 | 2004-2009 | Female | 60,008 | 40,669 | 59.8% (59.5% - 60.1%) |
|  |  | Male | 62,202 | 49,916 | 55.8% (55.5% - 56.1%) |
|  |  |  |  |  |  |
|  | 2010-2015 | Female | 72,176 | 40,804 | 64.1% (63.9% - 64.4%) |
|  |  | Male | 62,404 | 37,910 | 62.5% (62.2% - 62.8%) |
|  |  |  |  |  |  |
| 3 | 2004-2009 | Female | 42,016 | 14,320 | 44.9% (44.6% - 45.2%) |
|  |  | Male | 41,886 | 15,810 | 40.9% (40.6% - 41.2%) |
|  |  |  |  |  |  |
|  | 2010-2015 | Female | 52,279 | 15,059 | 50.1% (49.8% - 50.4%) |
|  |  | Male | 45,004 | 13,010 | 48.9% (48.6% - 49.2%) |
|  |  |  |  |  |  |
| 5 | 2004-2009 | Female | 35,941 | 3,996 | 40.5% (40.2% - 40.8%) |
|  |  | Male | 35,204 | 4,097 | 36.7% (36.4% - 37.0%) |
|  |  |  |  |  |  |
|  | 2010-2015 | Female | 36,113 | 4,025 | 46.0% (45.7% - 46.3%) |
|  |  | Male | 30,911 | 3,365 | 44.9% (44.6% - 45.3%) |
|  |  |  |  |  |  |
| 10 | 2004-2009 | Female | 28,165 | 2,934 | 37.0% (36.7% - 37.3%) |
|  |  | Male | 26,911 | 3,104 | 33.3% (33.0% - 33.5%) |
|  |  |  |  |  |  |
|  | 2010-2015 | Female | --------- | --------- | --------- |
|  |  | Male | --------- | --------- | --------- |
|  |  |  |  |  |  |
| 15 | 2004-2009 | Female | 3,058 | 591 | 35.6% (35.2% - 35.9%) |
|  |  | Male | 2,954 | 586 | 32.0% (31.7% - 32.3%) |
|  |  |  |  |  |  |
|  | 2010-2015 | Female | --------- | --------- | --------- |
|  |  | Male | --------- | --------- | --------- |
